# The E3 ligase TRIM7 suppresses the tumorigenesis of gastric cancer by targeting SLC7A11

**DOI:** 10.1038/s41598-024-56746-3

**Published:** 2024-03-20

**Authors:** Qishuai Chen, Tongtong Zhang, Runzhi Zeng, Kunmiao Zhang, Bingjun Li, Zhenguo Zhu, Xiaomin Ma, Yun Zhang, Linchuan Li, Jiankang Zhu, Guangyong Zhang

**Affiliations:** 1 Department of General Surgery, The First Affiliated Hospital of Shandong First Medical University, No.16766 Jingshi Road, Jinan, 250014 Shandong Province People’s Republic of China; 2 Department of Laboratory Medical, Zibo Central Hospital, Zibo, 255000 Shandong Province People’s Republic of China; 3 Department of General Surgery, Liaocheng People’s Hospital, Liaocheng, 252000 Shandong Province People’s Republic of China

**Keywords:** TRIM7, GC, SLC7A11, Ferroptosis, Ubiquitination, Gastric cancer, Clinical genetics, Oncogenesis

## Abstract

Tripartite motif-containing protein 7 (TRIM7), as an E3 ligase, plays an important regulatory role in various physiological and pathological processes. However, the role of TRIM7 in gastric cancer (GC) is still undefined. Our study detected the expression of TRIM7 in clinical specimens and investigated the regulatory effect and molecular mechanism of TRIM7 on GC progression through in vitro and in vivo experiments. Our finding showed that TRIM7 was significantly downregulated in GC, and patients with high expression of TRIM7 showed long overall survival. Both in vitro and in vivo experiments showed that TRIM7 dramatically suppressed the malignant progression of GC. Further investigation showed that ferroptosis was the major death type mediated by TRIM7. Mechanistically, TRIM7 interacted with SLC7A11 through its B30.2/SPRY domain and promoted Lys48-linked polyubiquitination of SLC7A11, which effectively suppressing SLC7A11/GPX4 axis and inducing ferroptosis in GC cells. In vivo experiments and correlation analysis based on clinical specimens further confirmed that TRIM7 inhibited tumor growth through suppressing SLC7A11/GPX4 axis. In conclusion, our investigation demonstrated for the first time that TRIM7, as a tumor suppressor, induced ferroptosis via targeting SLC7A11 in GC, which provided a new strategy for the molecular therapy of GC by upregulating TRIM7.

## Introduction

Gastric cancer (GC), the fifth most common malignancy worldwide, is the fourth leading cause of cancer-related death^1^. With the improvement of diagnosis and treatment, the prognosis of GC patients has improved^2^. Unfortunately, its recurrence and metastasis rates are still high^3^. Therefore, it is necessary to further elucidate the molecular mechanisms of progression, aiming to develop new molecular markers for its therapeutic strategies.

Tripartite motif-containing proteins (TRIMs) are a family of E3 ubiquitin ligases that mediate the degradation of endogenous proteins by the ubiquitin–proteasome system^4,5^. TRIMs have been reported to be related to the pathogenesis of various human malignancies including GC. For example, it has been previously reported that TRIM69 inhibits anoikis resistance and metastasis of GC through ubiquitin proteasome-mediated degradation of PRKCD^6^, and TRIM32 promotes GC cell proliferation and invasion by activating β-catenin signaling^7^.

TRIM7 contains an N-terminal RING finger, a B-Box domain, a Coiled coil domain and a C-terminal B30.2/SPRY domain^8^. It is dysregulated in a variety of human tumors, involving in regulating the proliferation of cancer cells such as glioblastoma, lung cancer, and osteosarcoma^9–11^. Specifically, TRIM7 can inhibit the growth of malignant tumors such as liver cancer, lung cancer, and kidney cancer^12–14^. However, the role of TRIM7 in the occurrence and progression of GC has not yet been reported.

Ferroptosis is a newly discovered cellular death^15^ that has been reported to effectively inhibit the progression of GC^16^. Solute Carrier Family 7 Member 11 (SLC7A11) and glutathione peroxidase 4 (GPX4) play crucial roles in the ferroptosis^17^. SLC7A11 inhibition could block the generation of cysteine and l-Glutathione (GSH), which subsequently triggered the accumulation of lipid peroxidation and eventually ferroptosis^15^. GPX4 is a key regulator of ferroptosis, which utilizes glutathione to convert lipid hydroperoxide into lipid alcohols. Inhibiting GPX4 weakens the anti-lipid peroxidation effect and induces ferroptosis^18^. Increasing evidence has confirmed that inhibiting the SLC7A11-GPX4 axis shows anti-cancer effects^19^. In this study, we firstly investigated the expression and function of TRIM7 in GC progression in an integrated system including clinical specimen detection, in vitro cellular experiments and in vivo animal experiments. Our investigation demonstrated for the first time that TRIM7 exerted anti-tumor effects through inducing ferroptosis via inhibiting SLC7A11-GPX4 axis.

## Materials and methods

### Samples

Thirty-eight pairs of GC tissues and matched adjacent tissues were obtained from GC patients received surgery for the treatment in The First Affiliated Hospital of Shandong First Medical University (Jinan, China). Each patient signed the informed consent. Tissue microarray (TMA) was purchased from Outdo Biotech (Cat. No.: HStmA180Sur-04; Shanghai, China). The methods of this study were reported in accordance with the Declaration of Helsinki. The study protocols were approved by the Ethical Committee of The First Affiliated Hospital of Shandong First Medical University (approval No.: 2021-S1039).

### Cell culture

AGS, HGC27 and HEK 293T cells were purchased from Procell Life Science and Technology (Wuhan, China). AGS cells were cultured on Ham’s F12K medium containing 10% fetal bovine serum (FBS, Thermo Fisher, CA, USA) and 1% penicillin/streptomycin (Thermo Fisher, CA, USA). HGC27 cells were cultured on minimum essential media (MEM, Gibco, Shanghai, China) containing 10% FBS and 1% penicillin/streptomycin. HEK 293T cells were cultured on DMEM containing 10% FBS and 1% penicillin/streptomycin. All cells were incubated in a humidified incubator in 5% CO_2_ at 37 °C. All cells were identified as mycoplasma free.

### Reagents

The utilized reagents in this study included ferrostatin-1 (cat. No.: S7243), Liproxstatin-1 (cat. No.: S7699), Z-VAD-FMK (cat. No.: S7023), Necrosulfonamide (cat. No.: S8251), 3-Methyladenine (cat. No.: S2767), MG132 (cat. No.: S2619), CHX (cat. No.: S7418) were purchased from Selleck Chemicals (Houston, Texas, USA). The stimulation concentration were as follows: Ferrostatin-1, 2 μΜ; Liproxstatin-1, 1 μM; Z-VAD-FMK, 10 μM; Necrosulfonamide, 0.5 μM; 3-Methyladenine, 250 μΜ; MG132, 10 μM; CHX 20 μM.

### Grouping

To investigate the effects of TRIM7 knockdown or overexpression on the cellular phenotypes, cells were divided into the following groups: (1) shTRIM7 group, transfected with short hairpin RNAs plasmid against TRIM7, and shNC group with scrambled sequences served as control; and (2) Flag-TRIM7 group, transfected with Flag-TRIM7 plasmid, and cells transfected using Flag-vector served as mock. All the procedures of transfection were conducted using the Lipofectamine 2000 (Thermo Fisher, CA, USA).

### CCK-8 assay and colony formation assay

The cells were utilized for the CCK-8 assay using commercial kit (Dojindo, Japan), according to the manufacturer’s instructions. Finally, the cellular viability was determined at a wavelength of 450 nm. For the colony formation assay, GC cell lines were incubated in 6-well plates and cultured for 14 days, followed by fixing with paraformaldehyde for 30 min. Subsequently, the cells were stained with 1% crystal violet (Solarbio, Beijing, China) for 15 min. The qualified colony were those with a diameter of more than 50 μm.

### Quantitative real-time PCR (qRT-PCR)

Total mRNA was extracted from tissues and GC cell lines using the RNeasy Mini kit (Qiagen, Hilden, Germany). The synthesis of cDNA was performed using PrimeScript RT kit (Takara, Dalian, China). The amplification was conducted using SYBR Green with the specific primers of TRIM7 (5′-GCTCGGGGTTGAGATCACC-3′; 5′-CCAGGCACATTGCTACACCT-3′) and SLC7A11 (5′-TCTCCA AAGGAGGTTACCTGC-3′; 5′-AGACTCCCCTCA GTA AAGTGAC-3′). GAPDH served as the internal standard. Finally, the results were evaluated using 2(− Delta Delta C(T)) method as previously described^20^.

### Western blot analysis

Total protein was extracted from tissues or cultured GC cells after washing twice with 1 × PBS and RIPA lysis on ice for 30 min. The protein sample (50 μg) was separated on an SDS-PAGE gel, then transferred to a PVDF membrane. Then the membrane was blocked in 5% BSA for 1 h at room temperature, and overnight at 4 °C with the corresponding primary antibody including TRIM7 (Proteintech, 26285-1-AP, 1:1000), SLC7A11 (Proteintech, 26864-1-AP, 1:1000), GPX4 (Proteintech, 67763-1-Ig, 1:1000), Flag (Proteintech, 66008-4-Ig, 1:1000), Myc (Proteintech, 16286-1-AP, 1:1000), HA (Proteintech, 81290–1-RR, 1:1000), GAPDH (Proteintech, 60004-1-Ig, 1:5000). After washing three times with PBST, the membrane was incubated with the corresponding secondary antibody for 1 h at room temperature. The blots were cut prior to hybridisation with antibodies during blotting. Finally, the protein bands were evaluated using the ImageJ software.

### Cell viability and cytotoxicity assay

GC cells (8 × 10^3^) were seeded into 96-well plates. After 48 h, cell viability was measured using CCK-8 kit (Dojindo, Japan) and the calcein/PI cell viability/toxicity detection kit (Beyotime, China). The cells were incubated with 10% CCK-8 solution at 37 °C for 2 h, and then the absorbance was measured at 450 nm. After incubation with calcein/PI buffer for 30 min, cellular viability was observed under a fluorescence microscope.

### Lipid peroxides measurement

Cells (1 × 10^5^) were washed twice with PBS, and incubated with 10 μM bodipy-581/591c11 (D3861, Thermo Fisher) at 37 °C in 5% CO_2_ for 30 min at room temperature. The liquid in the 12-well plates was discarded, followed by staining of nuclei using DAPI. The images were observed under a fluorescence microscope.

### Measurement of MDA and 4-HNE

Relative MDA concentrations in cell lysates were assessed using a lipid peroxidation (MDA) assay kit (S0131M, Beyotime, China) according to the manufacturer's instructions. The concentration of 4-HNE was assessed using an ELISA kit (MBS161454, MyBioSource, San Diego, CA, USA) according to the manufacturer's protocols.

### CoIP and LC–MS/MS

Cells were washed three times with PBS, and were scraped into lysis buffer containing protease inhibitors for 30 min, followed by centrifugation at 14,000 rpm for 20 min at 4 °C. Cell lysates were then added with the corresponding primary antibodies including Flag (Proteintech, 66008-4-Ig), Myc (Proteintech, 16286-1-AP), HA (Proteintech, 81290-1-RR), TRIM7 (Proteintech, 26285-1-AP), SLC7A11 (Proteintech, 26864-1-AP), and IgG (Cell Signaling Technology, #2729S), and incubated at 4 °C for 4 h, followed by incubation with protein A-agarose overnight at 4 °C. The above samples were separated by SDS-PAGE, and then were stained with Coomassie Brilliant Blue.

The tryptic peptides were sent to Jingjie BioLab (Hangzhou, China) for the LC–MS/MS, according to the previous description^21,22^. Briefly, the peptides were dissolved in 0.1% formic acid, and were then directly loaded onto a home-made reversed-phase analytical column. Afterwards, the peptides were subjected to NSI source followed by MS/MS in Q Exactive™ Plus (Thermo Fisher) coupled online to the UPLC. The voltage of electrospray was set at 2.0 kV. The m/z scan range was 350–1800 for full scan. The intact peptides were detected in the Orbitrap at a resolution of 70,000, and were selected for MS/MS using NCE setting as 28. The fragments were detected in the Orbitrap at a resolution of 17,500. A data-dependent procedure that alternated between one MS scan followed by 20 MS/MS scans with 15.0 s dynamic exclusion. Automatic gain control was set at 5E4.

### Immunofluorescence

Cells were fixed with prechilled methanol and permeabilized with 0.2% (v/v) Triton X-100 for 20 min at room temperature. Then the cells were blocked with 1% (v/v) BSA-PBS solution for 1 h at room temperature and then incubated with primary antibodies [Flag (66008-4-Ig, 1:1000), Myc (16286-1-AP, 1:400)] overnight at 4 °C. After washing three times with PBS, the secondary antibodies were diluted with 1% BSA-PBS solution and incubated for 1 h at room temperature. Then the nuclei were stained with DAPI, and finally the images were observed using a Carl Zeiss AG (GER) confocal microscope.

### Ubiquitination assay

For ubiquitination assay, Myc-SLC7A11 and HA-ubiquitin (HA-Ub) or HA-UB-48K, HA-UB-63K plasmids were transfected with or without Flag-TRIM7 using Lipofectamine 2000 transfection reagent. After 48 h, cells were lysed and analyzed by IP with anti-Myc antibody-conjugated agarose beads. After washing for 5 times, proteins were eluted from the beads and subjected to Western blot analysis.

### Induction of xenograft tumor model and grouping

BALB/c nude mice (6-week-old) purchased from Vital River (Beijing, China) were used for the xenograft tumor modeling. HGC27 cells with stable expression of Flag-vector or Flag-TRIM7 after lentiviral transfection were selected using puromycin, and were injected subcutaneously into the ipsilateral inguinal area of 20 nude mice. Upon availability of tumor on day 7, the animals were divided into 4 groups designated as Flag-vector + PBS group (n = 5), Flag-TRIM7 + PBS group (n = 5), Flag-TRIM7 + Myc-SLC7A11 group (n = 5), and Flag-TRIM7 + Fer-1 group (n = 5), respectively. The dose for Myc-SLC7A11 was 30 μg, while that for the Fer-1 was 10 mg/kg once per 3 days. The methods of animal studies were reported in accordance with the ARRIVE guidelines (https://arriveguidelines.org). The study procedures on animals were approved by the Ethical Committee of our hospital (2021-S199). All animal methods were carried out in accordance with relevant guidelines and regulations.

### Measurement of tumor volume and tumor weight

Approximately 7 days after treatment, tumor size was measured using vernier calipers. The calculation formula of tumor volume was based on the following formula: V = (length × width^2^)/2. On day 28, the animals were euthanized by intraperitoneal injection of sodium pentobarbital (200 mg/kg), followed by measuring the tumor volume and tumor weight. The tumor size was determined once per 3 days from day 7 to day 28.

### Immunohistochemistry (IHC)

IHC was performed using the tissue microarray and animal tissues. In brief, paraffin-embedded sections were deparaffinized in xylene and rehydrated in a graded alcohol series. Heat-activated antigen retrieval was performed using sodium citrate buffer (10 mmol/l, pH 6.0). Endogenous peroxidase activity was inhibited after 10 min of exposure to 3% hydrogen peroxide. Sections were then blocked with 5% BSA and incubated with the corresponding antibodies for 1 h at 37 °C, followed by incubating with HRP-conjugated secondary antibodies for 30 min at 37 °C. Sections were observed with DAB and microscopy with hematoxylin counterstaining.

### Statistical analysis

The experimental data were expressed as mean ± standard deviation from at least three independent replicates. For the comparison of data between two groups, an unpaired two-tailed Student's t-test was used. One-way or two-way ANOVA was utilized for the comparison of data from 3 or more groups. SPSS 23.0 software was used for statistical analyses. *P* values less than 0.05 were considered to be statistically significant.

## Results

### TRIM7 downregulation was associated with a poor prognosis in GC patients

**Figure 1:**
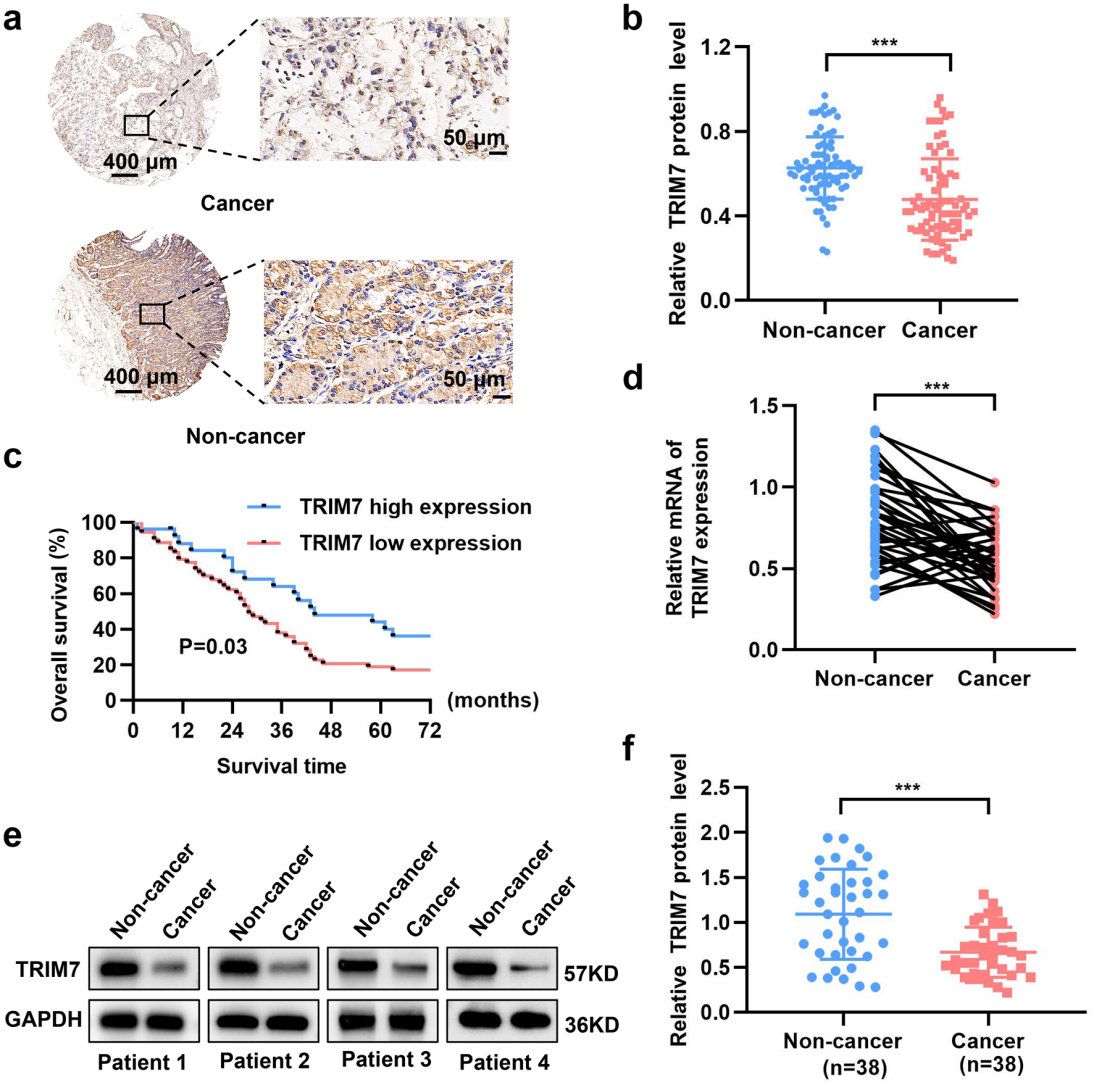
TRIM7 was down-regulated in GC tissues and was associated with poor prognosis. (**a**,**b**) Expression of TRIM7 protein in the GC tissues based on the IHC of the tissue microarray. (**c**) Low TRIM7 expression was associated with a poor prognosis in GC patients. (**d**) Expression of TRIM7 mRNA in the GC tissues and adjacent normal tissues obtained from the 38 GC patients. (**e**) Expression of TRIM7 protein in the GC tissues and adjacent normal tissues from 4 typical patients. (**f**) Relative TRIM7 protein expression in the paired samples of 38 GC patients. ***P < 0.001.

To investigate the expression of TRIM7 in GC, IHC was performed to analyze the expression of TRIM7 in 80 GC patients based on tissue microarray. As shown in Fig. 1a and b, the expression of TRIM7 in the GC tissues was significantly lower compared with that of adjacent tissues. Patients with low TRIM7 expression showed increased risk of high TNM stage and metastasis (Table 1). Kaplan–Meier analysis showed patients with low TRIM7 expression showed a poor overall survival (OS) compared with those with high TRIM7 expression (Fig. 1c). Consistently, the expression of TRIM7 mRNA and protein in the 38 GC patients admitted to our hospital was significantly lower in the cancer tissues compared with that of the adjacent tissues (Fig. 1d–f). Therefore, TRIM7 expression was significantly down-regulated in the GC tissues, and low TRIM7 expression was correlated with a poor OS.

**Table 1:** Clinicopathological characteristics and the relationship between TRIM7 expression in GC patients.

Characteristics	TRIM7 protein expression		*P* value
	Low (n = 54)	High (n = 26)	
Gender			0.742
Male	41 (75.9%)	22 (84.6%)	
Female	13 (24.1%)	4 (15.4%)	
Age, year	66.89	64.19	0.295
Tumor size (cm^3^, mean)	53.98	59.58	0.764
Lymph node metastasis			
N0	10 (18.5%)	11 (42.3%)	0.043
N1–N3	44 (81.5%)	15 (57.7%)	
TNM stage			0.018
I	0	2 (7.7%)	
II	20 (37%)	16 (61.5%)	
III	33 (61.1)	8 (30.8%)	
IV	1 (1.9%)	0	
Distant metastasis			
No	45 (83.3%)	25 (96.2%)	0.024
Yes	9 (16.7%)	1 (3.8%)	

### TRIM7 overexpression inhibited the proliferation of GC cells

**Figure 2:**
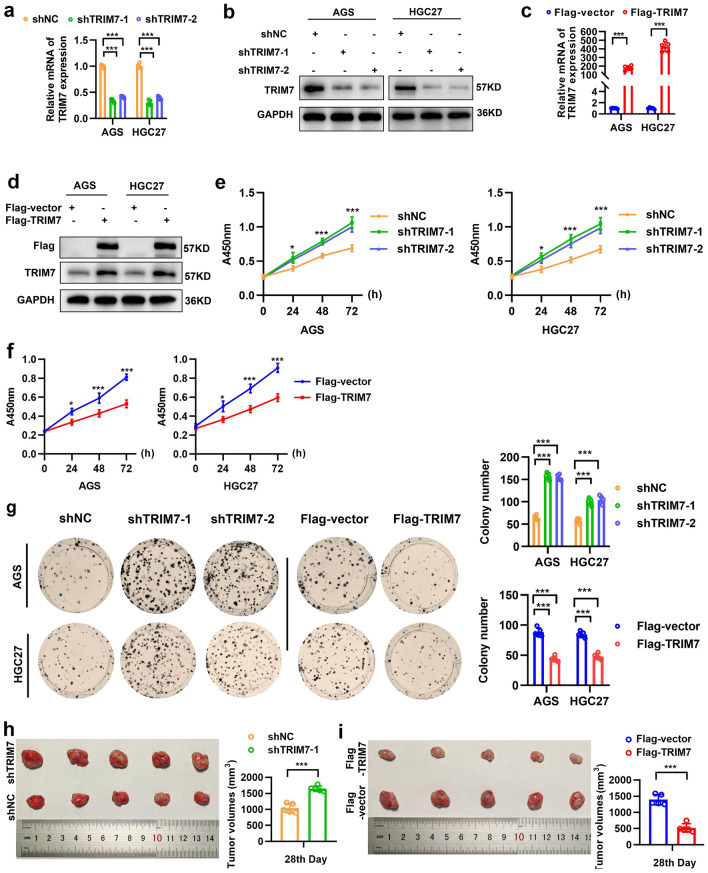
TRIM7 overexpression inhibited the proliferation of GC cells. (**a**–**d**) Both qRT-PCR and Western blot analysis confirmed the knockdown and overexpression of TRIM7 in cultured AGS and HGC27 cells. (**e**,**f**) Cell proliferation of GC cells in the presence of TRIM7 knockdown and overexpression based on CCK-8 assay. (**g**) Colony formation in the AGS and HGC27 cells in the presence of TRIM7 knockdown and overexpression. (**h**,**i**) Tumor volume in the mice received TRIM7 knockdown and overexpression on day 28. *P < 0.05; ***P < 0.001.

To further analyze the biological functions of TRIM7 in GC, we transfected AGS and HGC27 cells with plasmids to induce knockdown and overexpression of TRIM7. Then qRT-PCR and Western blot analysis were performed to validate the transfection efficiency respectively (Fig. 2a–d). CCK8 and colony formation assays confirmed that TRIM7 knockdown significantly enhanced the proliferation of GC cells. In addition, overexpression of TRIM7 significantly reduced the proliferation of GC cells (Fig. 2e–g). At the same time, we further studied the role of TRIM7 in vivo through subcutaneous tumor formation in nude mice. Animals with knockdown of TRIM7 showed significant increase in tumor volume (Fig. 2h). In contrast, the tumor volume in the animals with TRIM7 overexpression was significantly smaller compared with the control group (Fig. 2i). All these confirmed that TRIM7 could inhibit the proliferation of GC cells.

### TRIM7 overexpression promoted ferroptosis in GC cells

**Figure 3:**
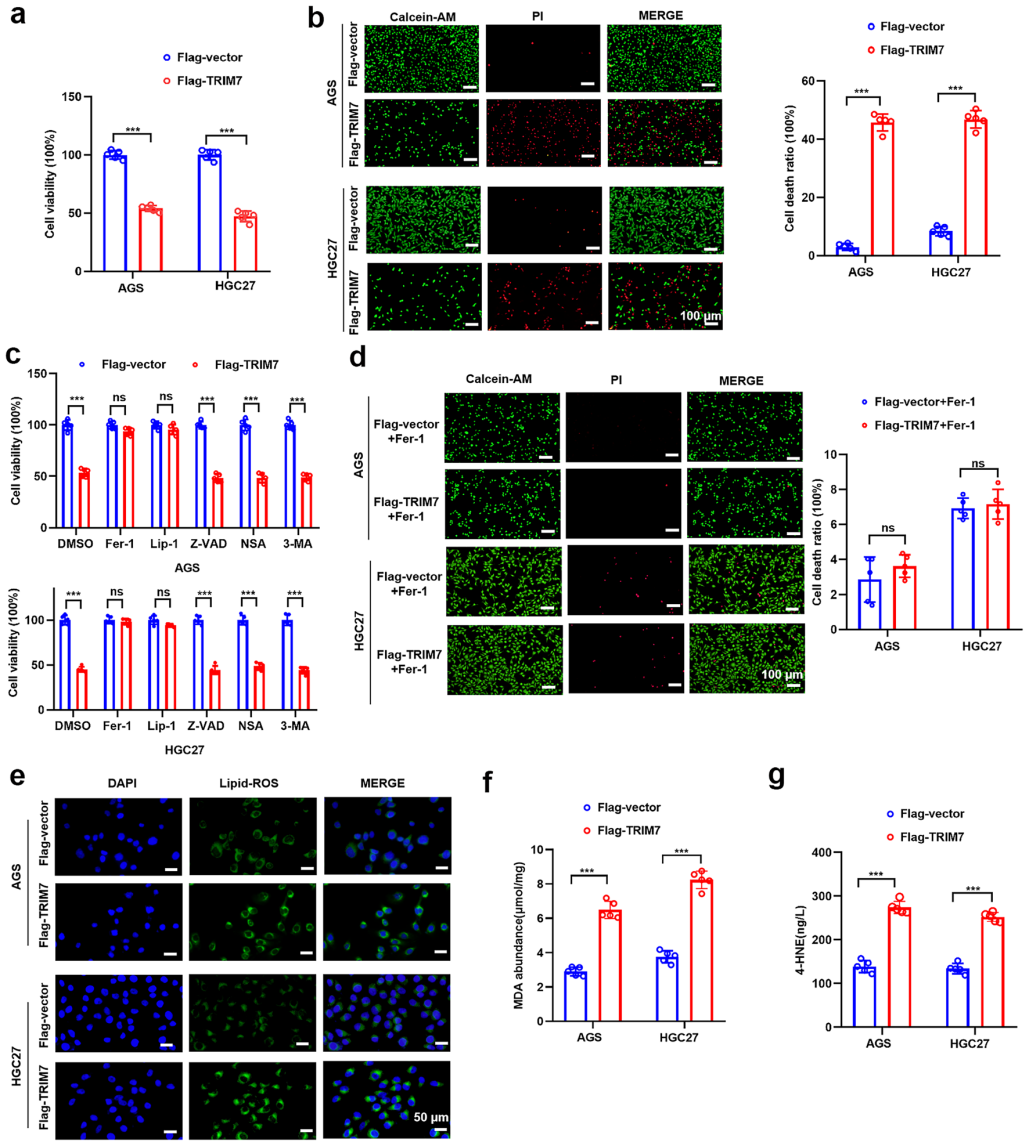
TRIM7 overexpression promoted the ferroptosis of GC cells. (**a**) TRIM7 overexpression reduced the cellular viability of GC cells. (**b**) Alive/dead cell viability assay showed that TRIM7 overexpression increased the cellular death. (**c**) Cellular death in TRIM7 overexpressing cells in the presence of different specific inhibitors such as Ferrostatin-1 (Fer-1), Liproxstatin-1 (Lip-1), Z-VAD-FMK (Z-VAD), Necrosulfonamide (NSA) and 3-Methyladenine (3-MA) at 48 h after transfection. (**d**) Alive/dead cell viability assay showed that cell death induced by overexpression of TRIM7 was reversed upon addition of the of the ferroptosis inhibitor Fer-1. (**e**) Lipid ROS in the TRIM7 overexpressing cells and control group after staining with BODIPY-C11. (**f**,**g**) Cellular MDA and 4-HNE expression in the TRIM7 overexpressing GC cells. ***P < 0.001; *ns* non-significant.

**Figure 4:**
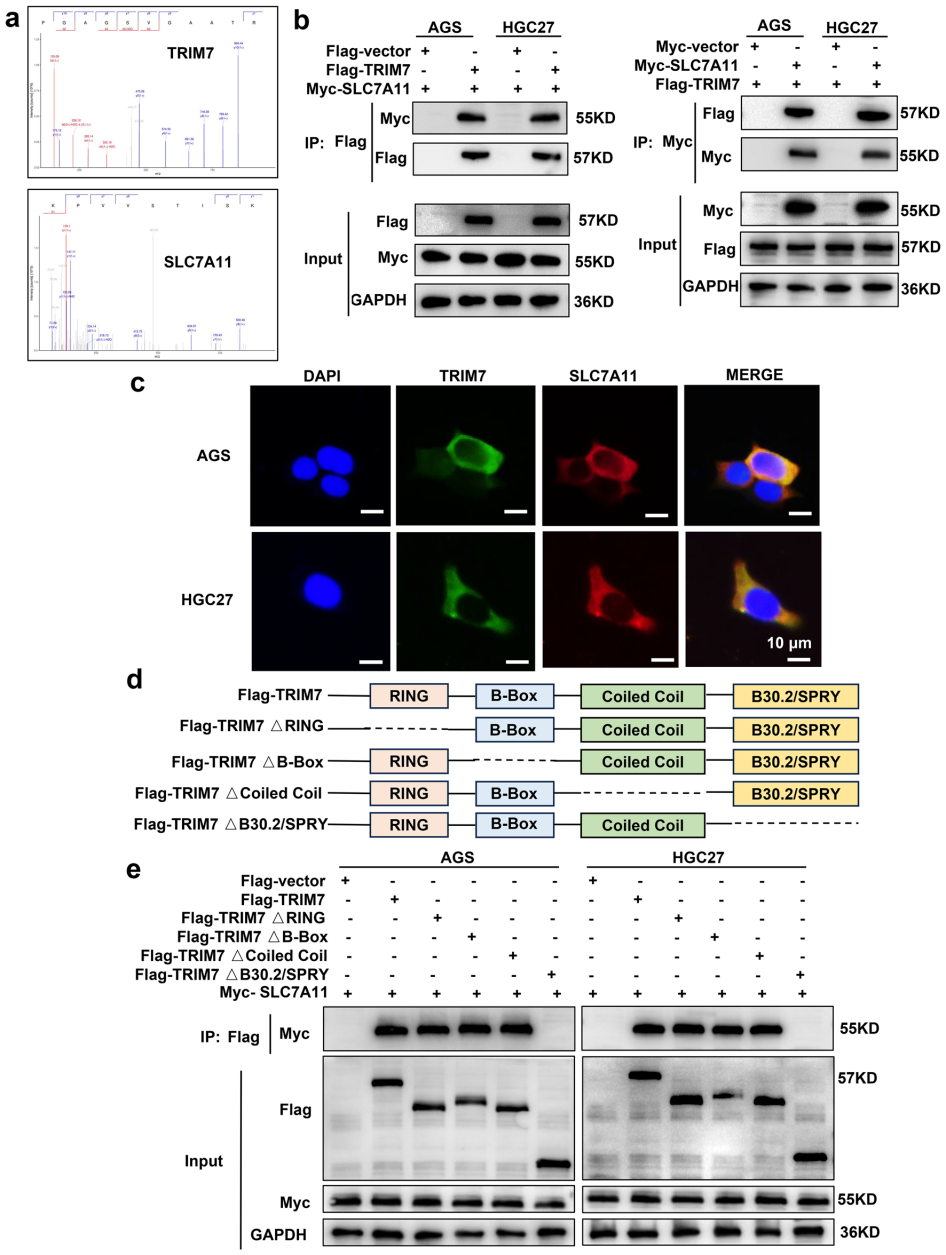
Interaction between TRIM7 and SLC7A11. (**a**) The secondary spectrum of the TRIM7 and SLC7A11 based on LC–MS/MS. (**b**) Exogenous protein interactions between TRIM7 and SLC7A11 in GC cells. (**c**) Colocalization of TRIM7 and SLC7A11 in GC cells based on immunofluorescence. (**d**) Schematic representation of wild type and truncation mutants of TRIM7. (**e**) After transfecting GC cells with the truncation mutant plasmid of TRIM7, Co-IP analysis was performed to measure the interaction between the TRIM7 truncation mutant and SLC7A11.

We evaluated cell death by detecting cell viability and cytotoxicity assay (Fig. 3a,b), which showed that overexpression of TRIM7 increased the cellular death of GC cells. Different specific inhibitors were utilized to further explore the exact type of GC cell death (e.g. apoptosis, necrosis, autophagy, and ferroptosis) caused by TRIM7 overexpression. Compared with the control group, TRIM7 overexpression-induced cell death was inhibited after treating with two independent ferroptosis inhibitors including Ferrostatin-1 (Fer-1) and Liproxstatin-1 (Lip-1) rather than the apoptosis inhibitor Z-VAD-FMK (Z-VAD), the necrosis inhibitor Necrosulfonamide (NSA) and the autophagy inhibitor 3-Methyladenine (3-MA) (Fig. 3c). Meanwhile, cell death caused by overexpression of TRIM7 was also reversed upon addition of the ferroptosis inhibitor Fer-1 (Fig. 3d).These results demonstrated that cell death caused by TRIM7 overexpression may be ferroptosis rather than apoptosis, necrosis or autophagy. To evaluate the effect of TRIM7 overexpression on cellular ferroptosis, we measured the expression of typical markers for the ferroptosis. BODIPY-C11 assay showed that overexpression of TRIM7 significantly increased the level of intracellular lipid peroxidation (Fig. 3e). Meanwhile, we found that the levels of MDA (Fig. 3f) and 4-HNE (Fig. 3g) were significantly increased in GC cells in the presence of TRIM7 overexpression. Therefore, our results showed that TRIM7 overexpression triggered the ferroptosis in these cells.

### TRIM7 interacted with SLC7A11

We then tried to elucidate the mechanism by which TRIM7 induced ferroptosis in GC. First, we transfected HEK 293-T cells with TRIM7-overexpressing and control plasmids. Then LC–MS/MS was utilized to select the possible molecular targets of TRIM7. We found that SLC7A11 was one of the candidate molecular targets of TRIM7 (Fig. 4a). To further verify the interaction between TRIM7 and SLC7A11, we performed exogenous and endogenous Co-IP verification in GC cells. Co-IP data showed that TRIM7 could interact with SLC7A11 (Fig. 4b, Supplementary Fig. S1a,b). Immunofluorescence of AGS and HGC27 cell lines confirmed intracellular colocalization of TRIM7 and SLC7A11 (Fig. 4c). TRIM7 consisted of 4 domains including RING (amino acid [AA] sequence 29-82), B-Box (AA sequence 125-166), Coiled Coil (AA sequence 166-263) and B30.2/SPRY (AA sequence 324-511) as shown in the protein structure analysis (UniProt: https://www.UniProt.org/). In order to study the molecular basis of the interaction between TRIM7 and SLC7A11, we subsequently constructed four truncated plasmids, named TRIM7ΔRING, TRIM7ΔB-Box, TRIM7ΔCoiled Coil and TRIM7ΔB30.2/SPRY, based on the above domain sequences and the wild-type (WT) plasmid of the complete TRIM7 sequence (Fig. 4d). To analyze the interaction domain between TRIM7 and SLC7A11, we then transfected the GC cells with these plasmids, respectively. The Co-IP results showed that TRIM7ΔRING, TRIM7ΔB-Box, and TRIM7ΔCoiled Coil mutants retained their interaction with SLC7A11. In contrast, the TRIM7ΔB30.2/SPRY mutant did not interact with SLC7A11 (Fig. 4e). All these indicated that TRIM7 interacted with SLC7A11 through the B30.2/SPRY domain.

### TRIM7 degraded SLC7A11 via ubiquitination

**Figure 5:**
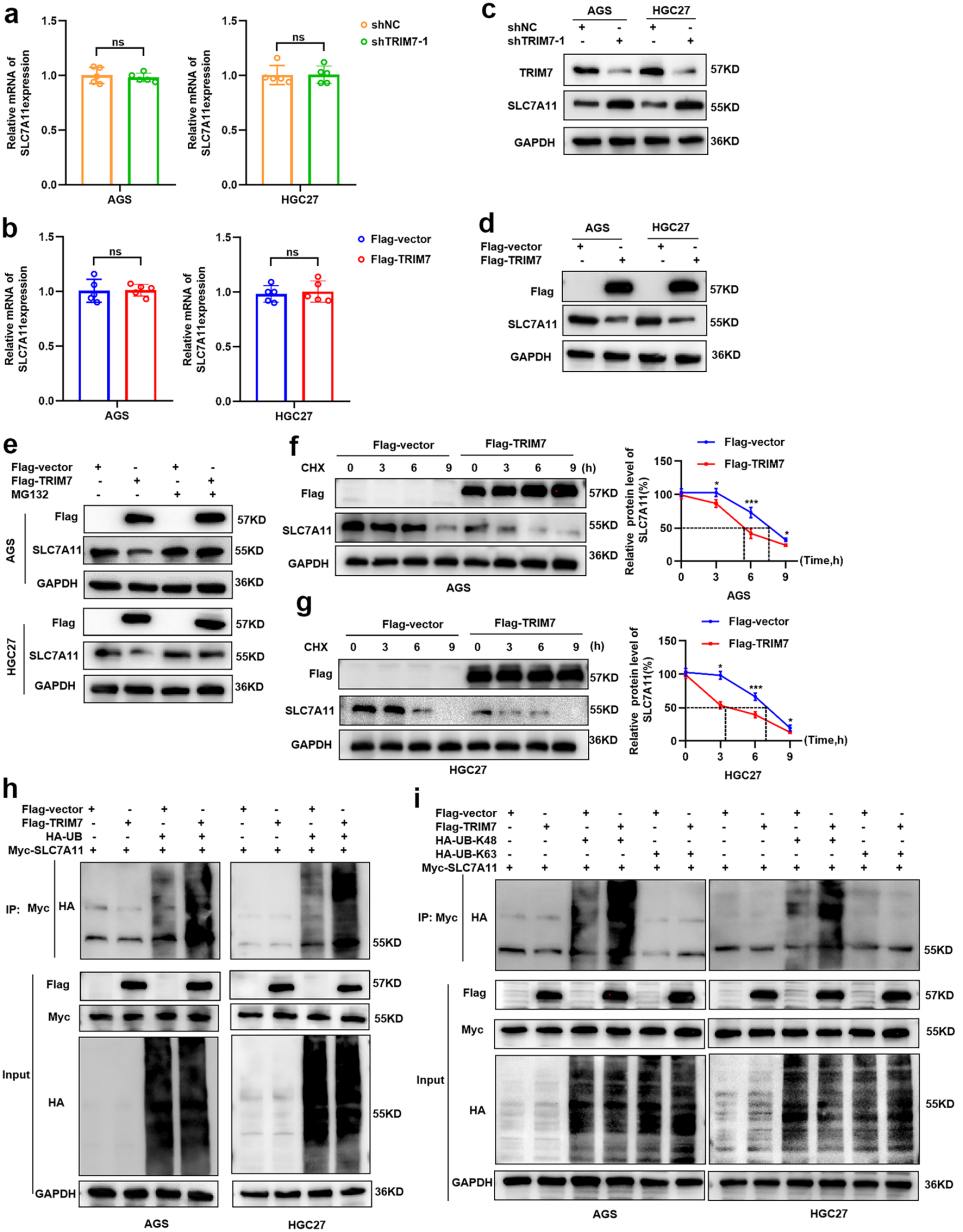
TRIM7 degraded SLC7A11 via ubiquitination. (**a**,**b**) Expression of SLC7A11 mRNA in the presence of TRIM7 knockdown or overexpression. (**c**,**d**) Expression of SLC7A11 protein in the presence of knockdown or overexpression plasmids and the corresponding mocks. (**e**) In GC cells overexpressing TRIM7 plasmid and control plasmid, the protein level of SLC7A11 was determined with or without MG132. (**f**,**g**) In GC cells overexpressing TRIM7 plasmid and control plasmid, cells were treated with cyclohexamide (CHX) for 0, 3 h, 6 h and 9 h, followed by determining the expression of SLC7A11. (**h**) Co-IP analysis of SLC7A11 ubiquitination in GC cells co-transfected with Flag-TRIM7 plasmid, Myc-SLC7A11 plasmid and HA-UB plasmid. (**i**) Co-IP analysis of SLC7A11 ubiquitination in GC cells co-transfected with Flag-TRIM7 plasmid, Myc-SLC7A11 plasmid and HA-UB-K48 or HA-UB-K63 plasmid. *P < 0.05; ***P < 0.001; *ns* non-significant.

In this section, we tested how TRIM7 regulated the expression of SLC7A11. As shown in Fig. 5a and b, TRIM7 knockdown and overexpression would not alternate the expression of SLC7A11 mRNA in both AGS and HGC27 cells. Western blot analysis showed significant upregulation of SLC7A11 after TRIM7 knockdown, and significant downregulation of SLC7A11 after TRIM7 overexpression (Fig. 5c,d). Moreover, TRIM7 was verified to interact with SLC7A11 through its B30.2/SPRY domain, and TRIM7 did not degrade SLC7A11 after B30.2/SPRY deletion (Supplementary Fig. S2a). Besides, the effects of TRIM7 on GC cell proliferation inhibition (Supplementary Fig. S2b,c) and ferroptosis induction (Supplementary Fig. S2d–f) were effectively reversed in the absence of B30.2/SPRY domain. To further investigate the mechanism of SLC7A11 degradation, we treated the GC cells with a specific proteasome inhibitor MG132. The results showed that the downregulation of SLC7A11 induced by high expression of TRIM7 could be reversed after treating with MG132 (Fig. 5e). After blocking de novo protein synthesis in the TRIM7-overexpressing cells by cycloheximide (CHX), TRIM7 could significantly promote the downregulation of SLC7A11 (Fig. 5f,g). Western blot analysis showed that TRIM7 overexpression could significantly increase the SLC7A11 ubiquitination (Fig. 5h). These data indicated that TRIM7 degraded SLC7A11 protein expression through proteasome-mediated ubiquitination. After proving that TRIM7 ubiquitination mediated SLC7A11 degradation, we further sought the type of polyubiquitination of SLC7A11. To date, K48- and K63-linked chains have been well acknowledged as the two most abundant chain types^23,24^. Our data showed that TRIM7 promoted Lys48 (K48)-linked but not Lys63 (K63)-linked polyubiquitin chain to SLC7A11 protein (Fig. 5i). This implied that SLC7A11 polyubiquitination occurred primarily in the form of K48-linked ubiquitin chains. Taken together, these data indicated that TRIM7 induced K48-linked SLC7A11 polyubiquitination.

### TRIM7 activated ferroptosis and inhibited tumor progression by inhibiting the SLC7A11/GPX4 axis

**Figure 6:**
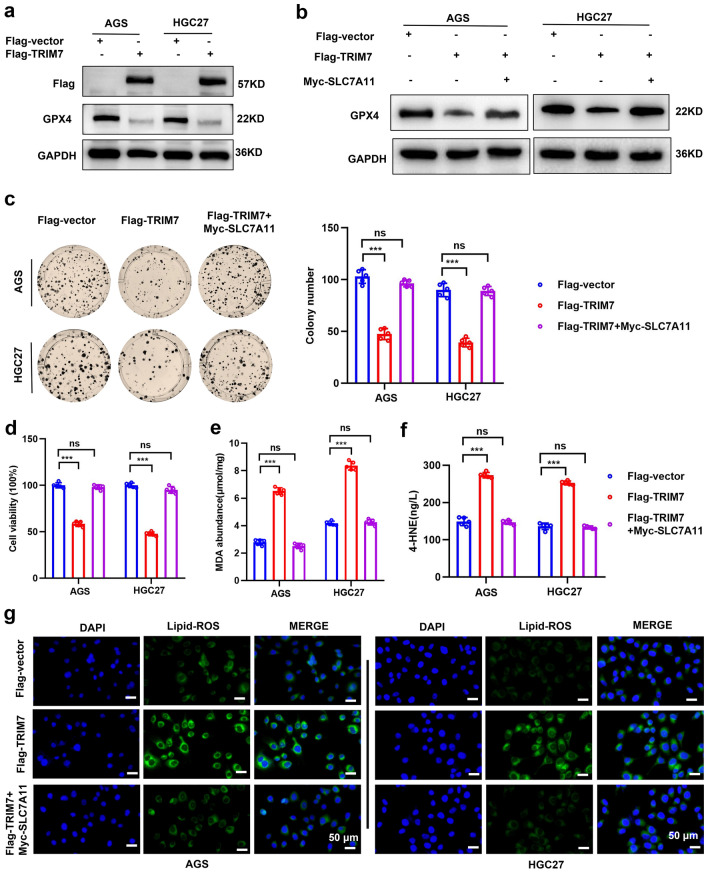
TRIM7 activated ferroptosis and suppressed tumor progression by inhibiting the SLC7A11/GPX4 axis. (**a**) Western blot analysis of the protein expression level of GPX4 in GC cells transfected with Flag-TRIM7 plasmid. (**b**) Protein expression level of GPX4 in GC cells transfected with Flag-TRIM7 plasmid and simultaneously transfected with Myc-SLC7A11 plasmid. (**c**,**d**) The colony formation test and cell viability test of GC cells transfected with Flag-TRIM7 plasmid and simultaneously transfected with Myc-SLC7A11 plasmid. (**e**,**f**) Expression of MDA and 4-HNE in GC cells transfected with Flag-TRIM7 plasmid and simultaneously transfected with Myc-SLC7A11 plasmid. (**g**) Fluorescent image of BODIPY-C11 staining after GC cells were transfected with Flag-TRIM7 plasmid and simultaneously transfected with Myc-SLC7A11 plasmid. ***P < 0.001; *ns* non-significant.

Increasing evidence indicates that SLC7A11 is a ferroptosis suppressor gene that is overexpressed in many human cancers^25^. In addition, it is a member of the cystine/glutamate antiporter protein, which is used to uptake cysteine for glutathione biosynthesis and anti-aging agent defense^26^. GPX4 mediates the conversion of toxic lipid peroxides into nontoxic lipid alcohols in the presence of GSH^27^. Inhibition of SlC7A11 may lead to GSH depletion and subsequent reduction in GPX4 expression, which ultimately results in cellular/subcellular membrane injuries caused by iron-dependent accumulation of lipid peroxides^28^. Based on these studies, we speculated that TRIM7 may activate ferroptosis by regulating the SLC7A11/GPX4 axis, thereby inhibiting tumor progression. Initially, we determined the expression of GPX4 after overexpressing TRIM7. In the presence of TRIM7 overexpression in GC cells, the protein expression of GPX4 was down-regulated (Fig. 6a). This was reversed in the presence of SLC7A11 overexpression (Fig. 6b). When treating TRIM7 overexpressing GC cells with a plasmid overexpressing SLC7A11, the decreased proliferation and increased cell death caused by TRIM7 overexpression were reversed (Fig. 6c,d), together with the reversal of ferroptosis-related indices (i.e. increased cellular lipid peroxidation, MDA, and 4-HNE) (Fig. 6e–g). The above evidence suggested that TRIM7 activated ferroptosis and inhibited tumor progression by inhibiting the SLC7A11/GPX4 axis.

### TRIM7 effectively inhibited the growth of xenograft tumors in nude mice

**Figure 7:**
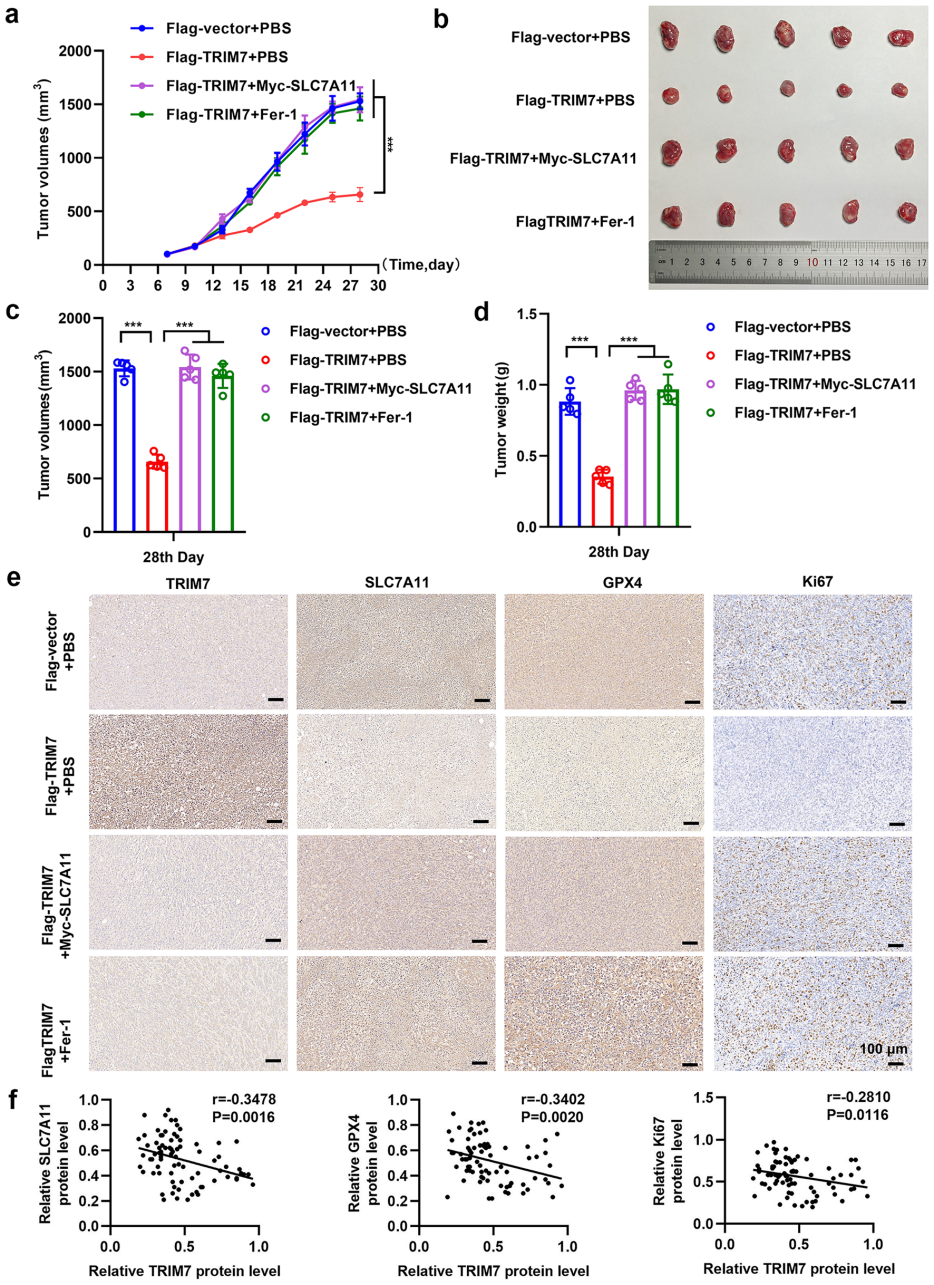
TRIM7 effectively inhibited the growth of xenograft tumors in nude mice. (**a**) The tumor volume in different groups from day 7 to day 28. (**b**) Macroscopic images of tumors in different groups on day 28 after cell injection. (**c**,**d**) Tumor volume and weight in different groups on day 28. (**e**) Representative images of IHC of TRIM7, SLC7A11, GPX4, and Ki67 in different groups. (**f**) Correlation analysis of TRIM7 and SLC7A11, GPX4, or Ki67 expression on tissue microarray. ***P < 0.001.

The tumor growth curve showed that compared with the control group, the tumor growth rate of the Flag-TRIM7 group was significantly slower from day 7 to day 28 (Fig. 7a), while that of the Myc-SLC7A11 group and the ferroptosis inhibitor group showed no statistical difference compared with that of the control group. The tumor volume and tumor weight were significantly reduced in the Flag-TRIM7 group compared with those of the other three groups (Fig. 7b–d). IHC staining verified that the expression of Ki67, SLC7A11 and GPX4 was significantly reduced in the TRIM7 overexpression group (Fig. 7e). In order to verify the relationship between TRIM7 and the SLC7A11/GPX4 axis in clinical specimens, we performed IHC staining on 80 GC tissue microarray. As shown in Fig. 7f, the expression of SLC7A11, GPX4 and Ki67 was negatively related to TRIM7 expression in GC tissues. These data were consistent with the in vitro experimental data and further verified the tumor suppressive effects of TRIM7 by inhibiting the SLC7A11/GPX4 axis.

## Discussion

As an E3 ubiquitin ligase, TRIM7 is highly expressed in certain malignancies, which promotes the proliferation of cancer cells, such as osteosarcoma and glioblastoma^10,11^. In contrast, its low expression could inhibit the proliferation of liver cancer cells^14^. In this study, we first confirmed that the expression of TRIM7 was lower in GC tissues compared with the adjacent tissue, and patients with low TRIM7 expression showed a poor prognosis. Then, in vitro experiments were performed based on knockdown and overexpression of TRIM7, which confirmed that TRIM7 knockdown promoted GC cell proliferation, while its overexpression can inhibit GC cell proliferation and induce GC cell death. Our data showed that the inhibition of proliferation induced by TRIM7 overexpression was associated with ferroptosis rather than apoptosis, necrosis or autophagy. For the mechanism, our data showed that TRIM7 could induce K48-linked polyubiquitination of SLC7A11, which then resulted in the inhibition of its downstream GPX4. Our study provides helpful information on the research and development of anti-GC drugs.

As a special type of post-translational modifications, ubiquitination plays crucial roles in regulating the function of proteins via modulating the protein degradation. Protein ubiquitination process mainly involves ubiquitin-activating enzyme (E1), ubiquitin conjugating enzyme (E2) and substrate-specific ubiquitin ligase (E3)^29^. These enzymes promote the attachment of the mono-Ub or Ub chains to the substrate proteins in a sequential manner. K48 along with K11- and K29-linked polyubiquitin chains could mark the proteins that would be degraded by proteasome, while the K63-linked multiple mono-ubiquitin and poly-ubiquitination would involve in the regulation of lysosomal pathways^30^. As a family member of TRIMs, TRIM7 constitutes a small fraction of the RING-type E3 ligase protein family, which mediates the ubiquitination of different substrates by interacting with the E2 ubiquitin-binding enzymes^8^.

In a previous study, TRIM7 was reported to regulate the tumorigenesis and chemoresistance in osteosarcoma through BRMS1 ubiquitination^10^. Many previous studies have confirmed that the TRIMs family can affect tumor progression by regulating ferroptosis^31,32^. SLC7A11 and GPX4 are important regulators of ferroptosis^33^. SLC7A11 is a cellular transmembrane protein that can transport extracellular cystine into cells for cysteine production and glutathione biosynthesis, and combat cellular oxidative stress by maintaining cellular levels of GSH and inhibition of ferroptosis. Multiple studies have also confirmed the importance of the SLC7A11-GSH-GPX4 axis in ferroptosis^33,34^. In this study, LC–MS/MS validated that SLC7A11 was the target of TRIM7, and their interaction were proved by Co-IP and colocalization test. TRIM7 could directly bind with SLC7A11 through targeting the C-terminal B30.2/SPRY domain. This indicated that TRIM7 could induce the polyubiquitination of K48-linked polyubiquitination of SLC7A11, further leading to the inhibition of its downstream GPX4. This confirmed that the TRIM7-SLC7A11-GPX4 axis involved in the progression of GC and played a role in inhibiting the growth of GC.

There are really some limitations in this study. Although we validated that TRIM7 induced the degradation of SLC7A11 and the subsequent degradation of GPX4, we did not investigate the GSH changes in the presence of TRIM7. Nevertheless, this would not hamper the accuracy of our results, as many previous studies have confirmed that SLC7A11-GSH-GPX4 axis plays a very important role in regulating ferroptosis^35,36^.

In summary, we identified TRIM7 as a novel negative regulator of SLC7A11. TRIM7 induced K48-linked polyubiquitination of SLC7A11 protein, further leading to the inhibition of its downstream GPX4 and acting as a tumor suppressor in GC. Thus, this study may contribute to the research and development of new strategies for the treatment of GC.

### Supplementary Information

Supplementary Figures.

## Data Availability

The datasets used and/or analysed during the current study are available from the corresponding author on reasonable request.
