# Supplementary material for: The E3 ligase TRIM7 suppresses the tumorigenesis of gastric cancer by targeting SLC7A11
Source: Sci Rep. 2024 Mar 20;14:6655. doi: 10.1038/s41598-024-56746-3 (PMC10954695; doi:10.1038/s41598-024-56746-3)

Supplementary data

The E3 ligase TRIM7 suppresses the tumorigenesis of gastric cancer by targeting SLC7A11

Qishuai Chen[1], Tongtong Zhang[2], Runzhi Zeng[1], Kunmiao Zhang[3], Bingjun Li[1], Zhenguo Zhu[1], Xiaomin Ma[1], Yun Zhang[1], Linchuan Li[1], Jiankang Zhu[1], Guangyong Zhang[1, *]

[1] Department of General Surgery, The First Affiliated Hospital of Shandong First Medical University, Jinan 250014, Shandong Province, People's Republic of China.

[2] Department of Laboratory Medical, Zibo Central Hospital, Zibo 255000, Shandong Province, People's Republic of China.

[3] Department of General Surgery, Liaocheng People’s Hospital, Liaocheng 252000, Shandong Province, People’s Republic of China.

[*] **Correspondence:** Guangyong Zhang, Department of General Surgery, The First Affiliated Hospital of Shandong First Medical University, No.16766 Jingshi Road, Jinan 250014, Shandong Province, People's Republic of China. Email: guangyongzhang@hotmail.com

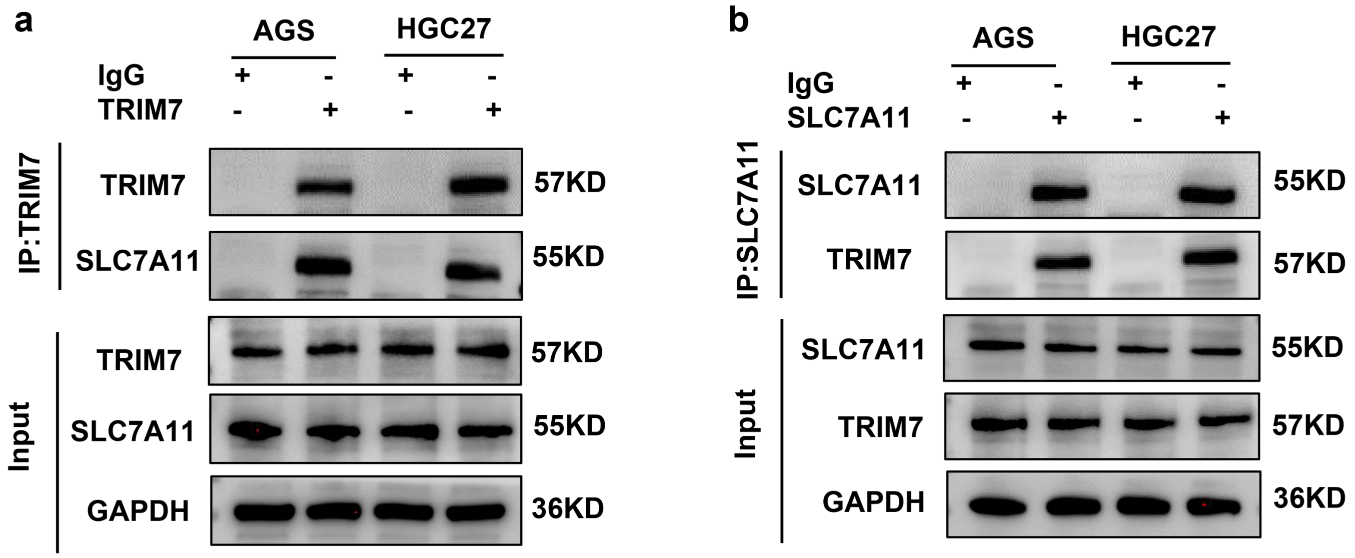

Supplementary Figure 1 Interaction between TRIM7 and SLC7A11. (a, b) Endogenous protein interactions between TRIM7 and SLC7A11 in GC cells.

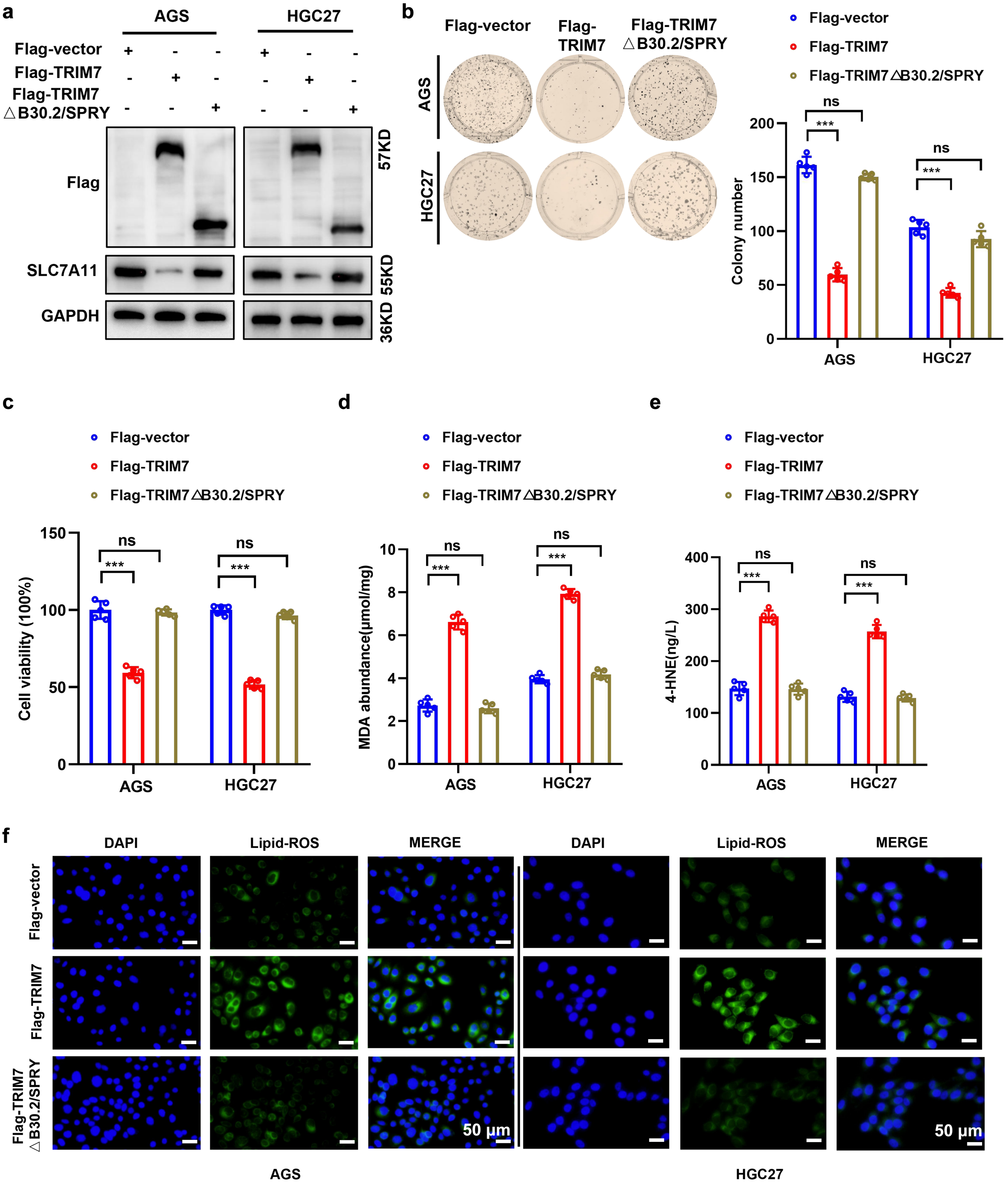

Supplementary Figure 2 TRIM7 degraded SLC7A11 via structural domain B30.2/SPRY and exerted an inhibitory effect on GC cells. (a) Protein expression level of SLC7A11 in GC cells transfected with Flag-TRIM7 plasmid and Flag-TRIM7△B30.2/SPRY plasmid. (b, c) The colony formation test and cell viability test of GC cells transfected with Flag-TRIM7 plasmid and Flag-TRIM7△B30.2/SPRY plasmid. (d, e) Expression of MDA and 4-HNE in GC cells transfected with Flag-TRIM7 plasmid and Flag-TRIM7△B30.2/SPRY plasmid. (f) Fluorescent image of BODIPY-C11 staining of GC cells after transfecting with Flag-TRIM7 plasmid and Flag-TRIM7△B30.2/SPRY plasmid. ***$P < 0.001$; ns, non-significant.

Catalogue numbers of the **ladders 1**:

Multicolor Prestained Protein Ladder, WJ 103(10kDa~250kDa), Shanghai Epizyme Biomedical Technology Co., Ltd,Shanghai,China

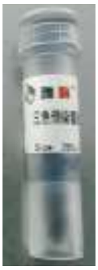

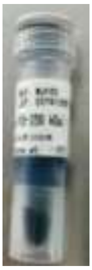

Catalogue numbers of the **ladders 2**:

number：26616,Thermo Scientific™, MA,USA,product number：26616

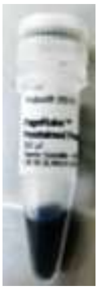

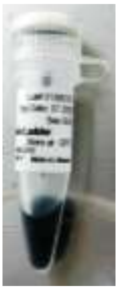

Model of the **machine 1** on which the picture was exposed：

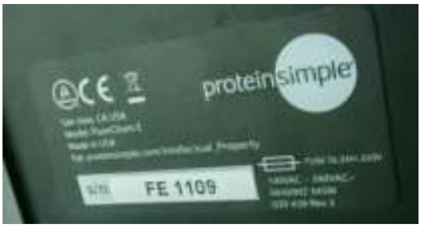

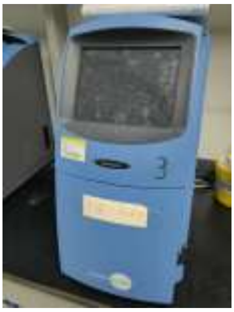

Model of the **machine 2** on which the picture was exposed：

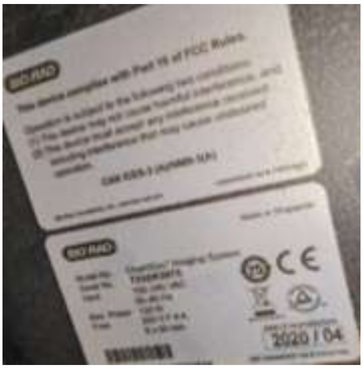

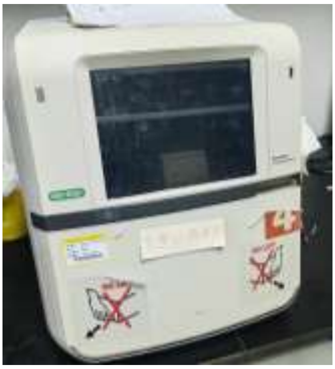

Figure 1e

on fig：

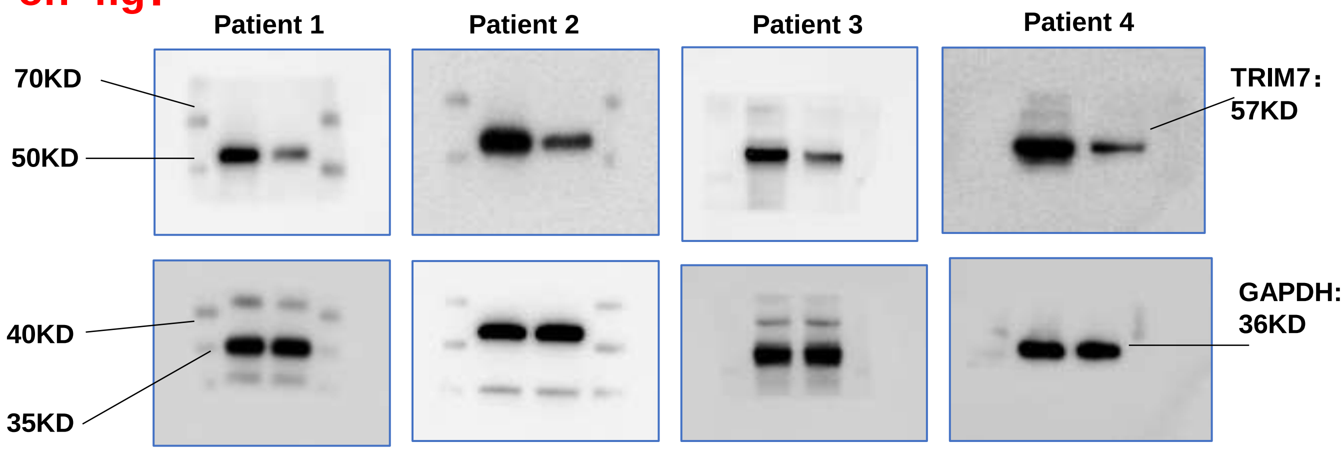

Repeat：

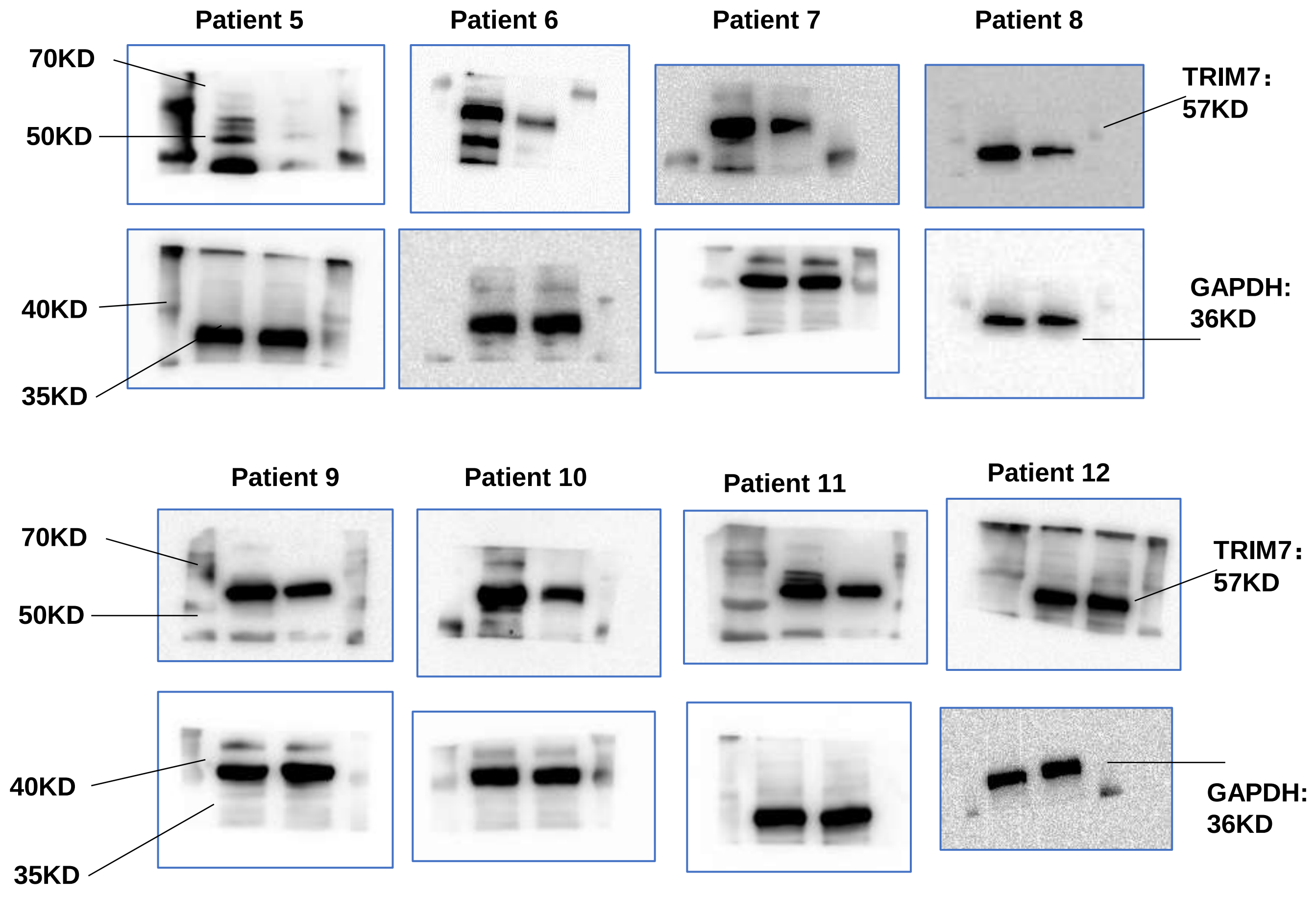

Figure 1e

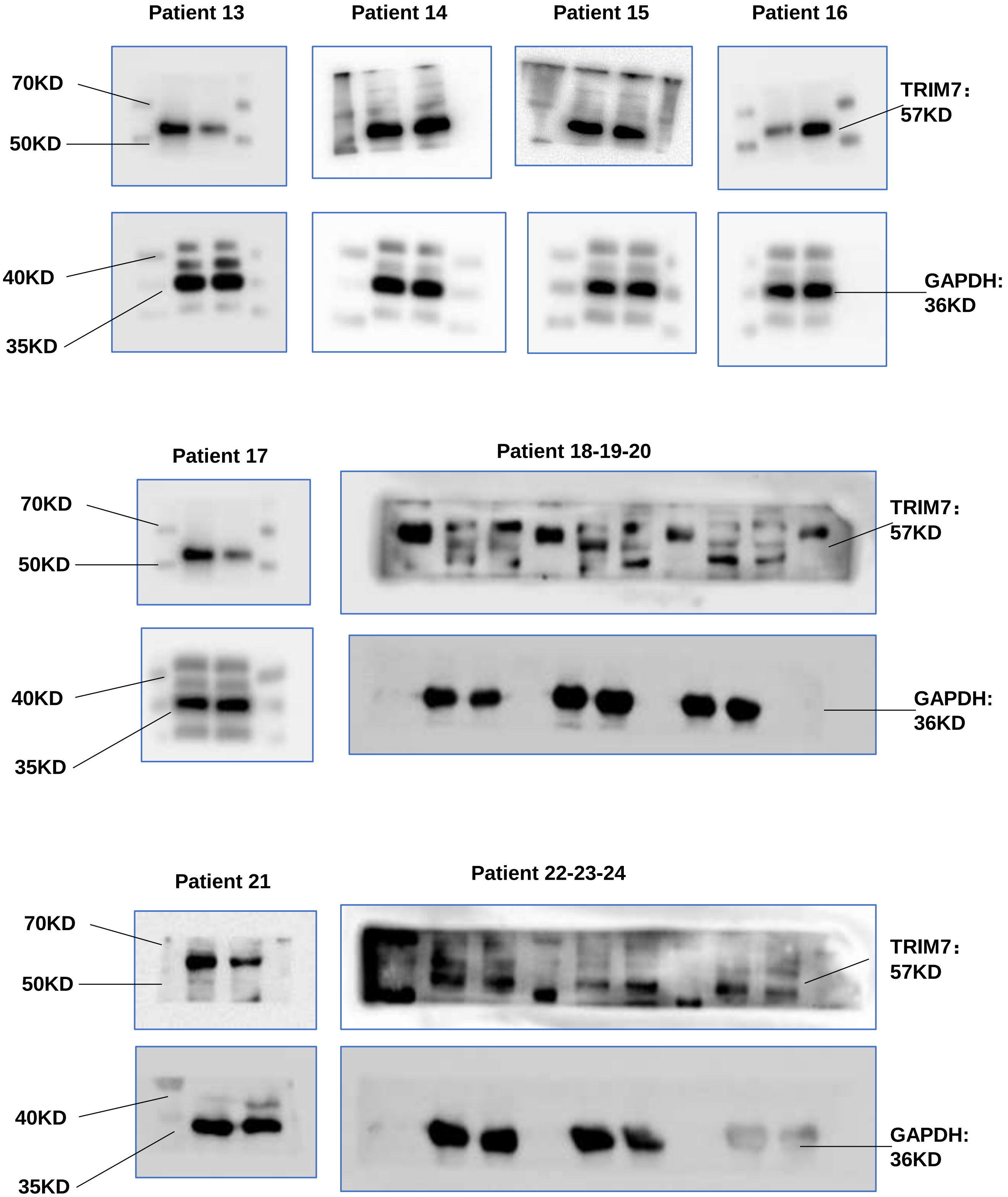

Patient 13
Patient 14
Patient 15
Patient 16
70KD
50KD
TRIM7:
57KD
40KD
35KD
GAPDH:
36KD
Patient 17
Patient 18-19-20
70KD
50KD
TRIM7:
57KD
40KD
35KD
GAPDH:
36KD
Patient 21
Patient 22-23-24
70KD
50KD
TRIM7:
57KD
40KD
35KD
GAPDH:
36KD

Figure 1e

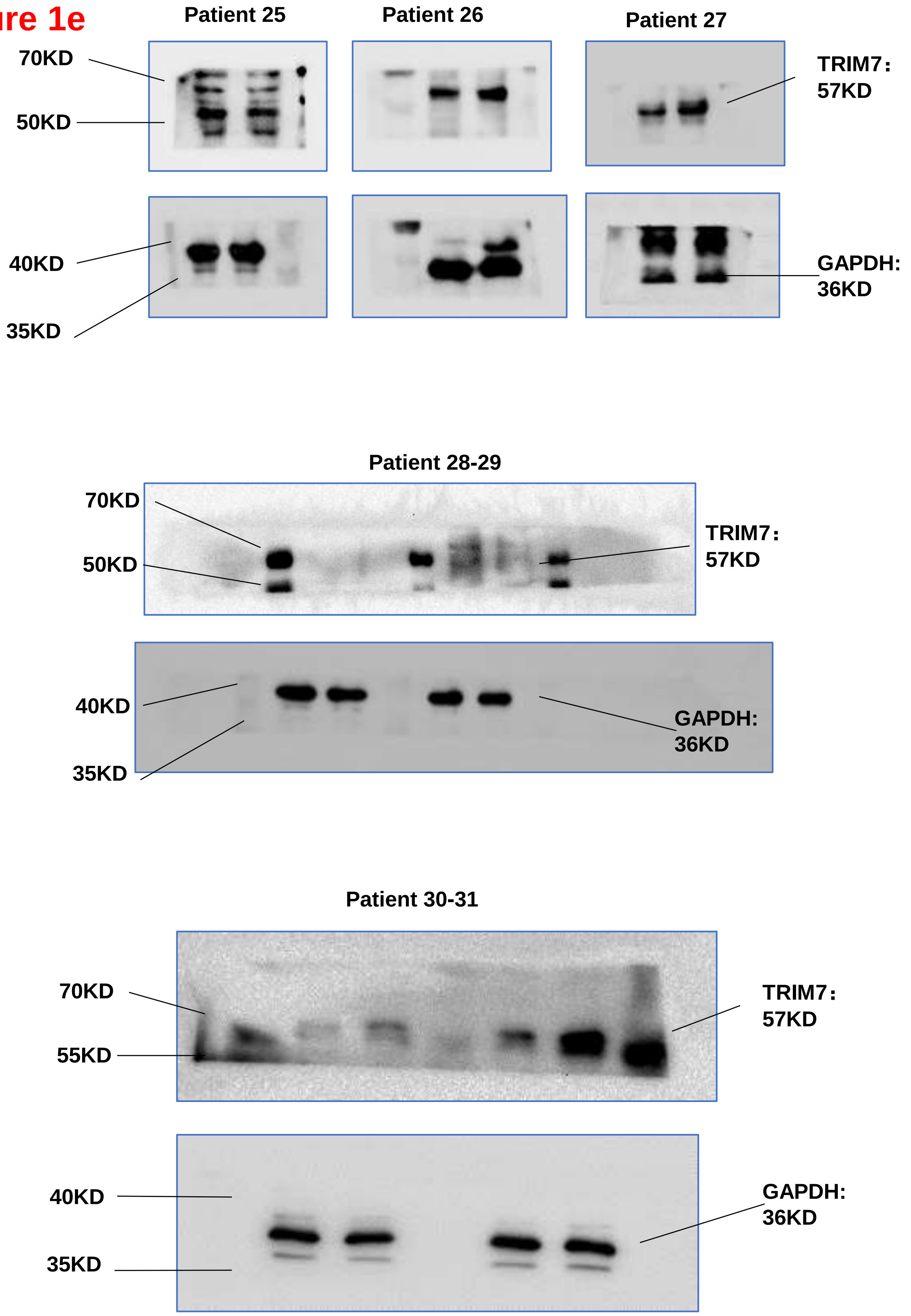

Patient 25
Patient 26
Patient 27
70KD
50KD
TRIM7：
57KD
40KD
35KD
GAPDH:
36KD
Patient 28-29
70KD
50KD
TRIM7：
57KD
40KD
35KD
GAPDH:
36KD
Patient 30-31
70KD
55KD
TRIM7：
57KD
40KD
35KD
GAPDH:
36KD

Figure 1e

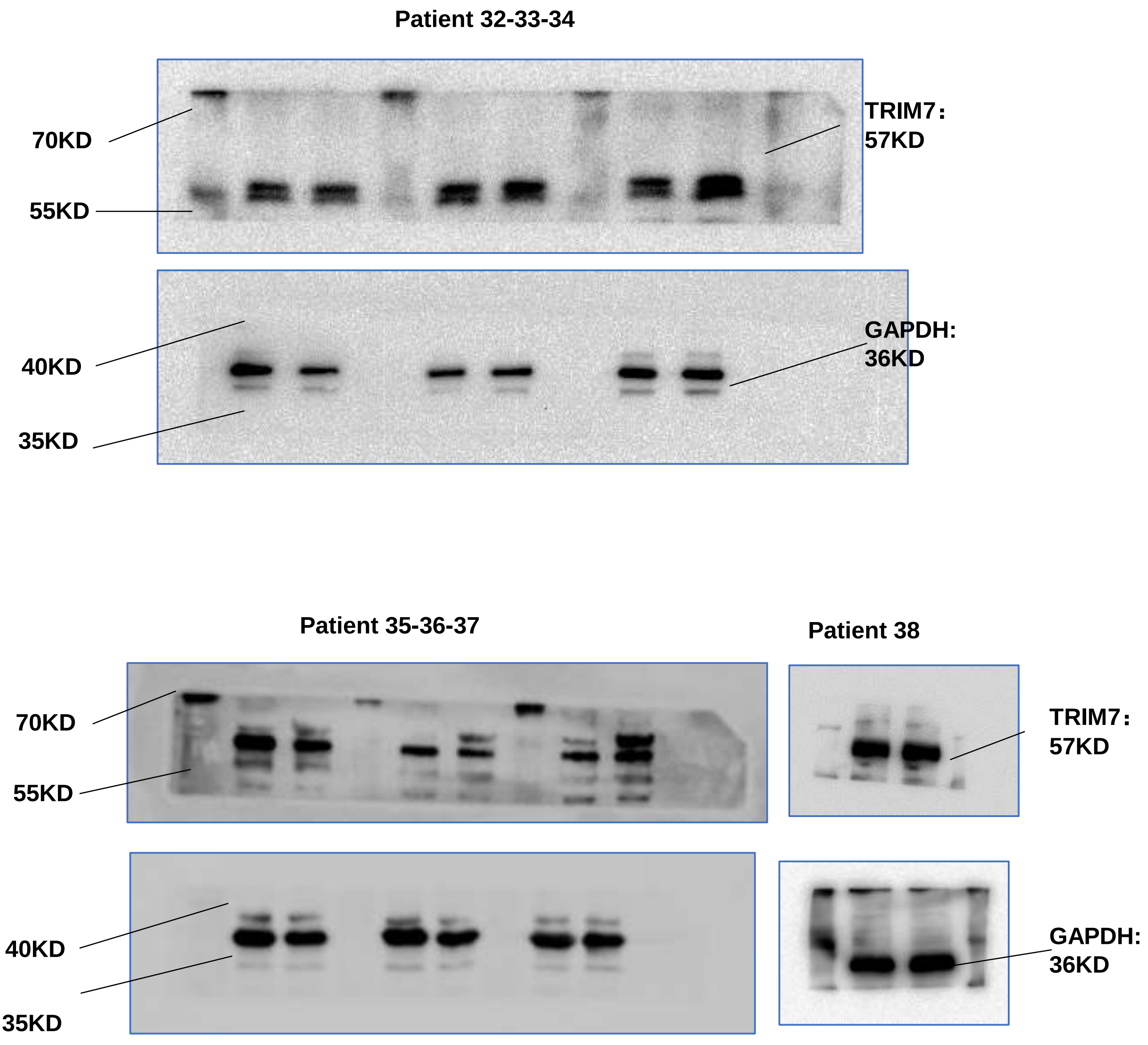
Patient 32-33-34
70KD
55KD
TRIM7：
57KD
40KD
35KD
GAPDH:
36KD
Patient 35-36-37
Patient 38
70KD
55KD
TRIM7：
57KD
40KD
35KD
GAPDH:
36KD

Figure 2.b

on fig：

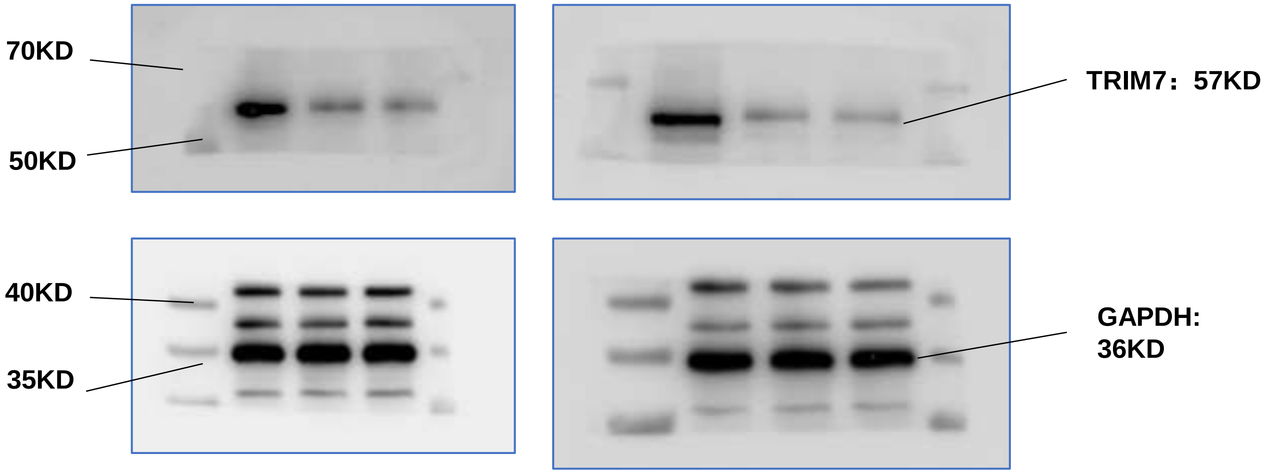

Repeat：

1：

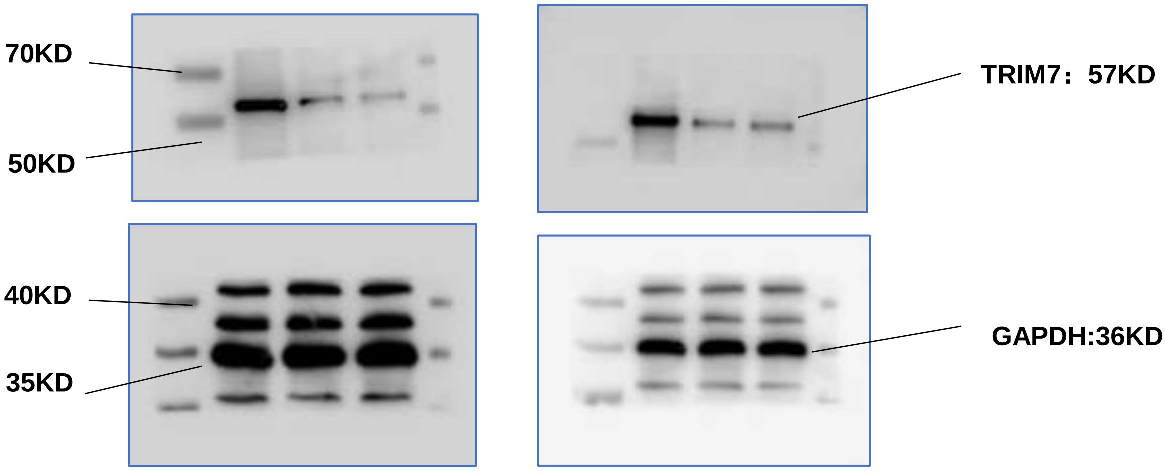

2：

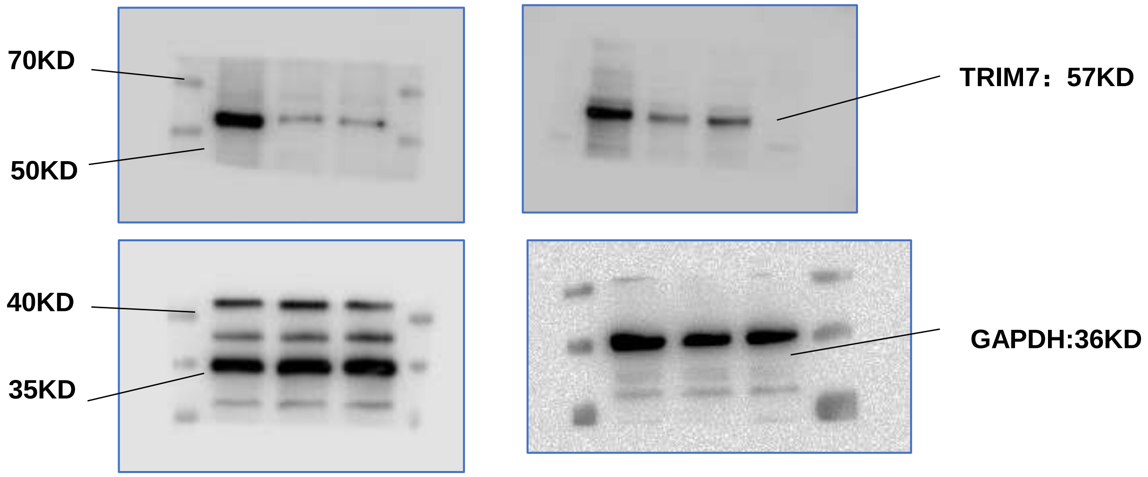

Figure 2d

on fig：

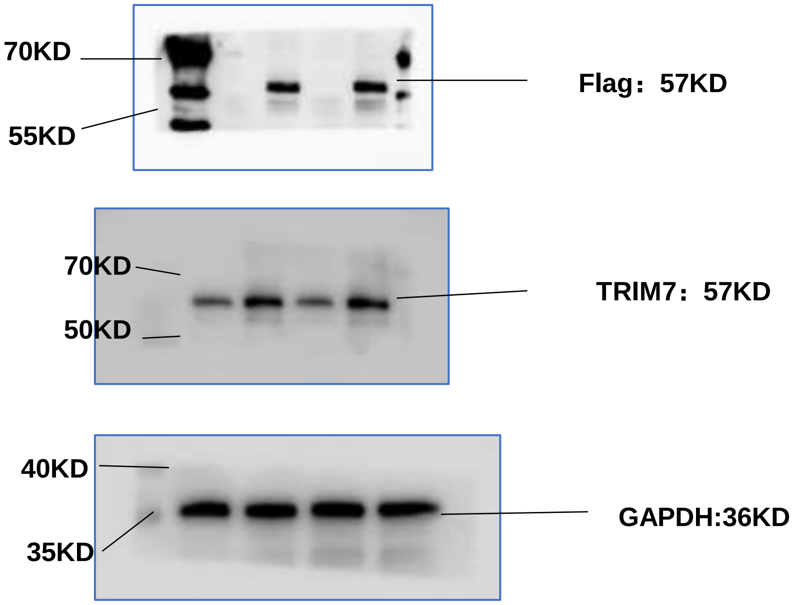

Repeat：

1：

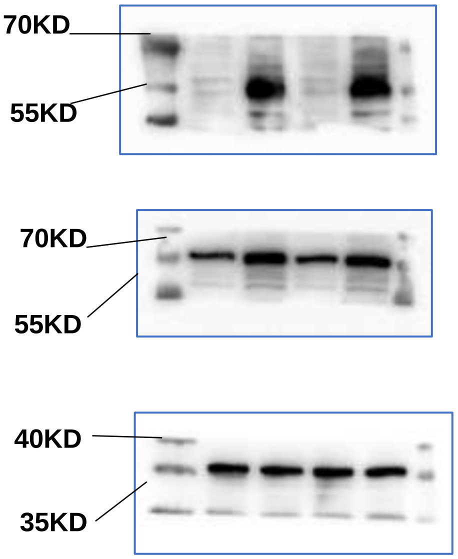

2：

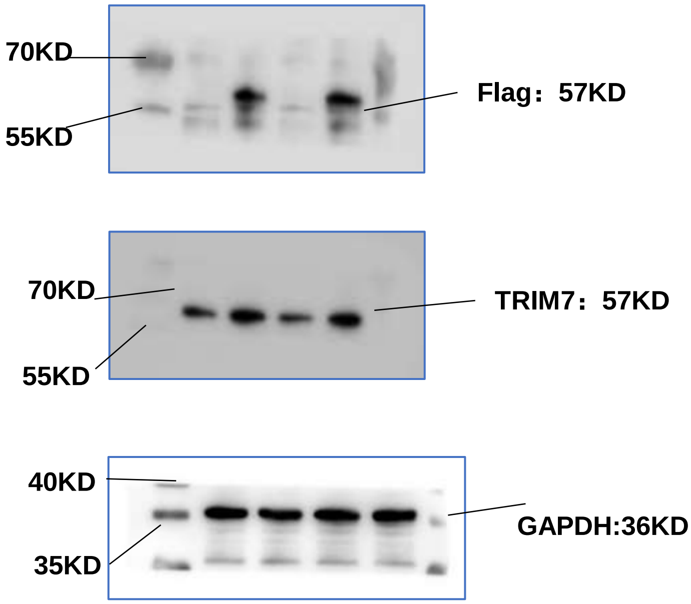

Figure 4b

on fig (left):

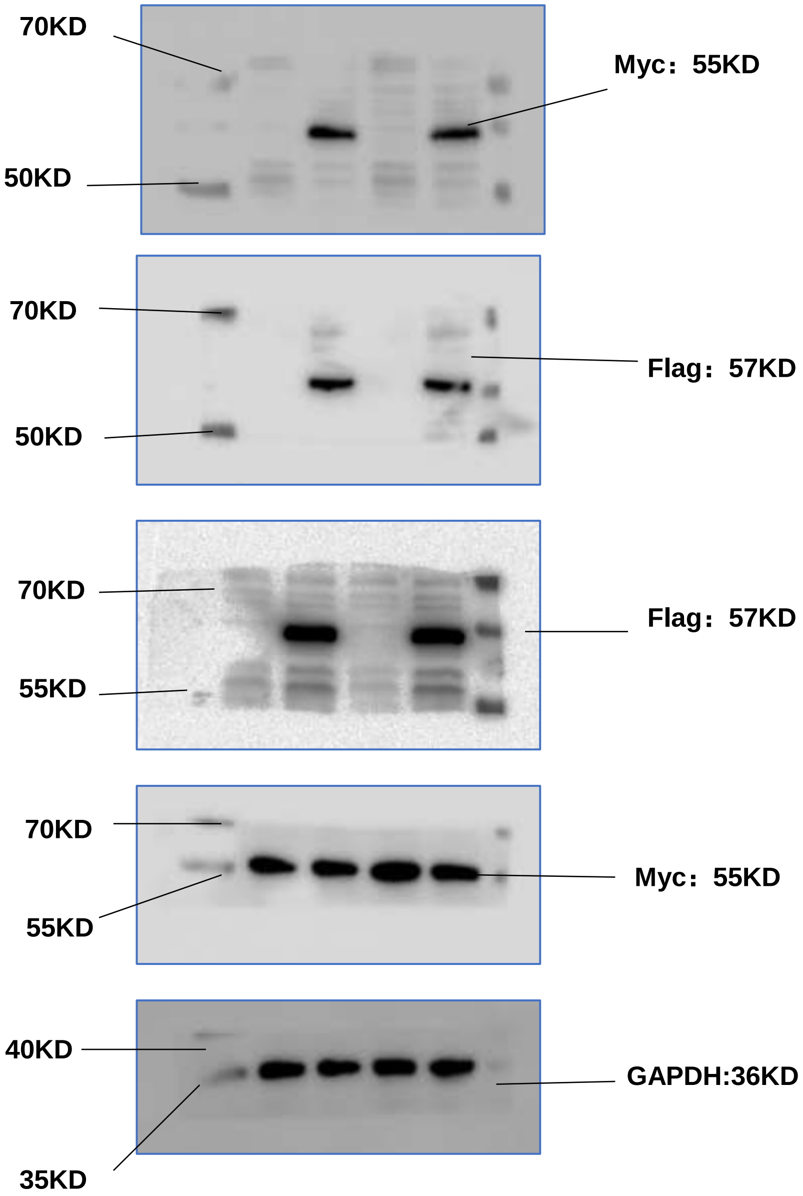

Figure 4b

Repeat（left）：

1：

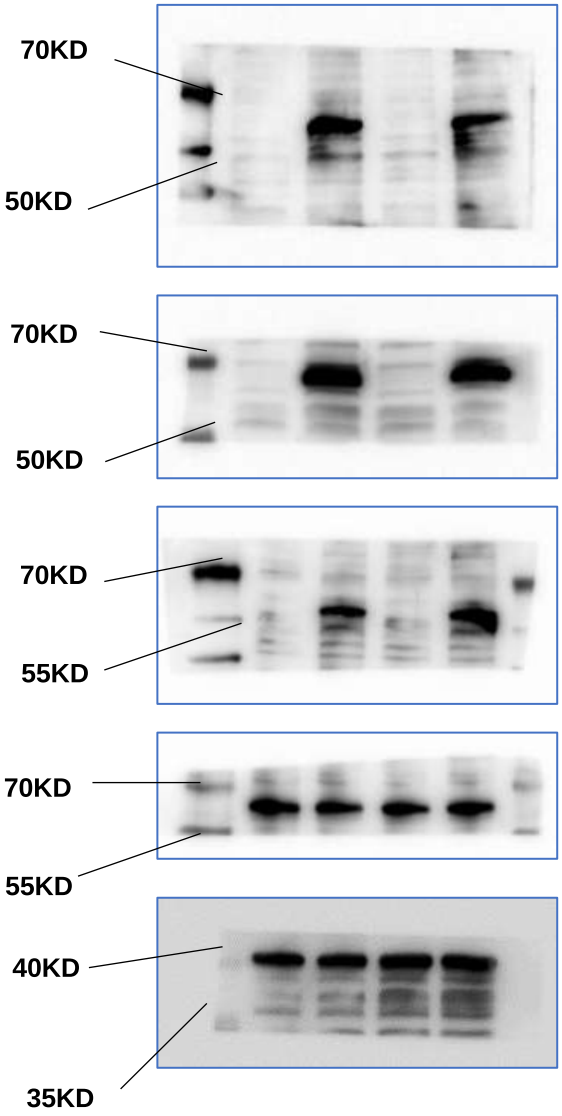

2：

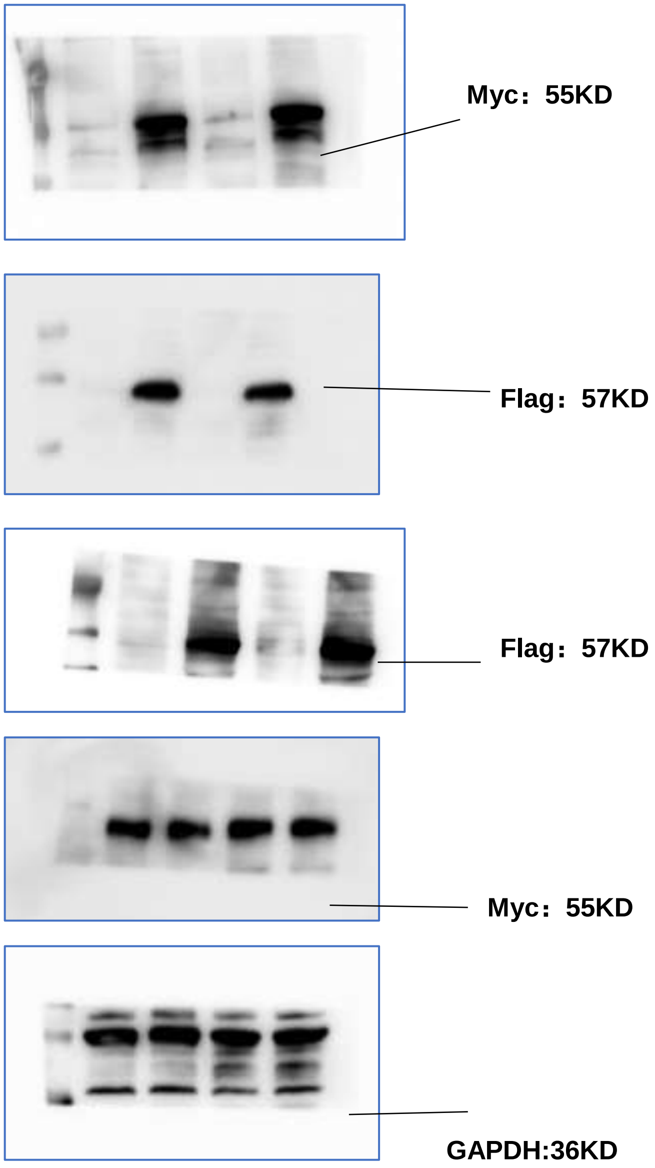

Figure 4b

on fig (right)：

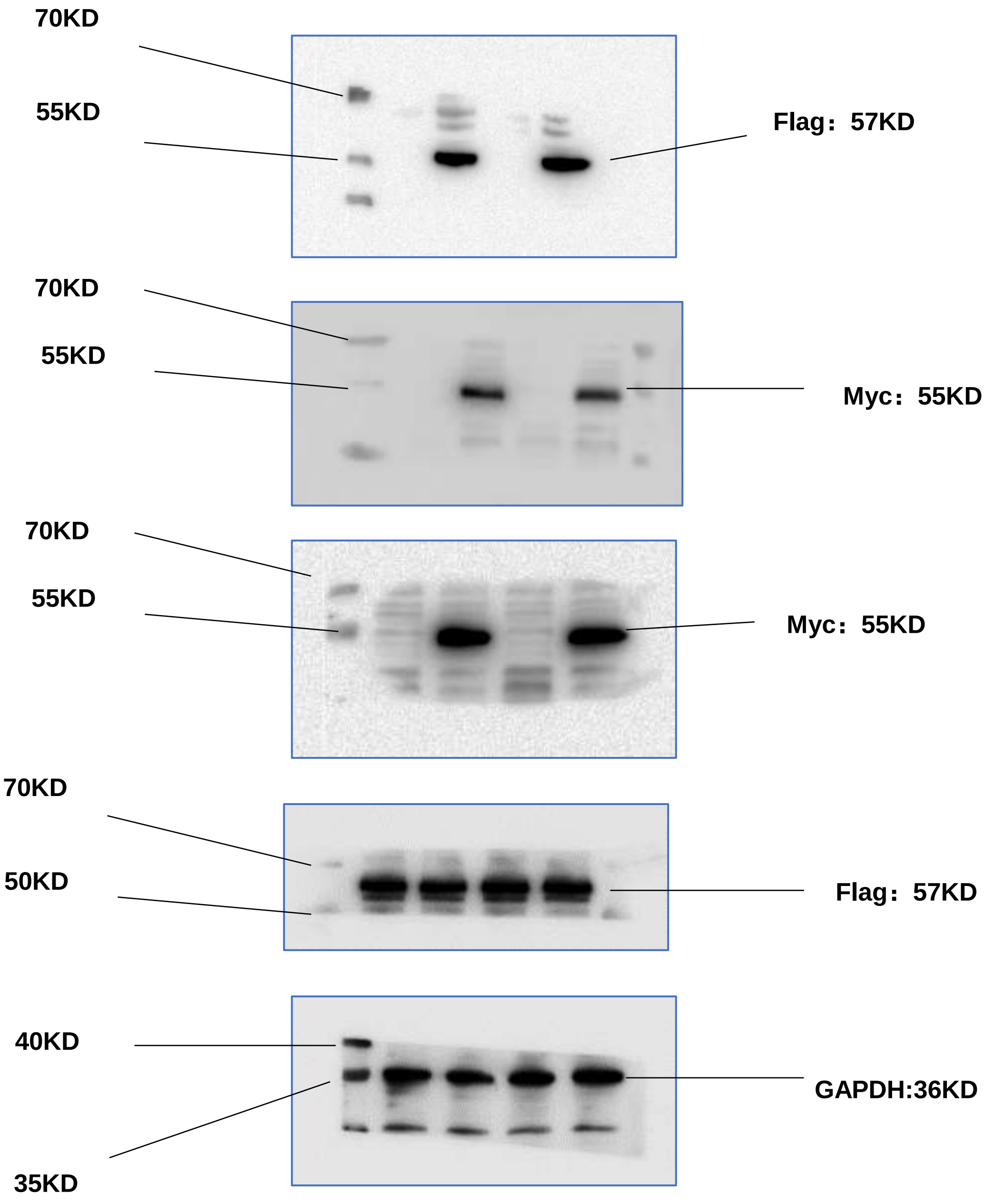

Figure 4b

Repeat（right）：

1：

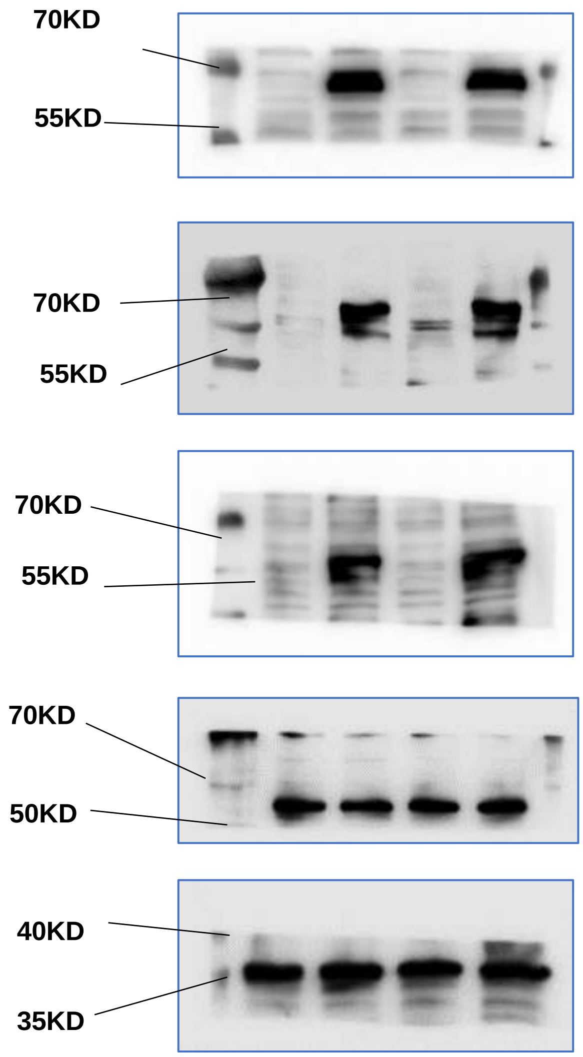

2：

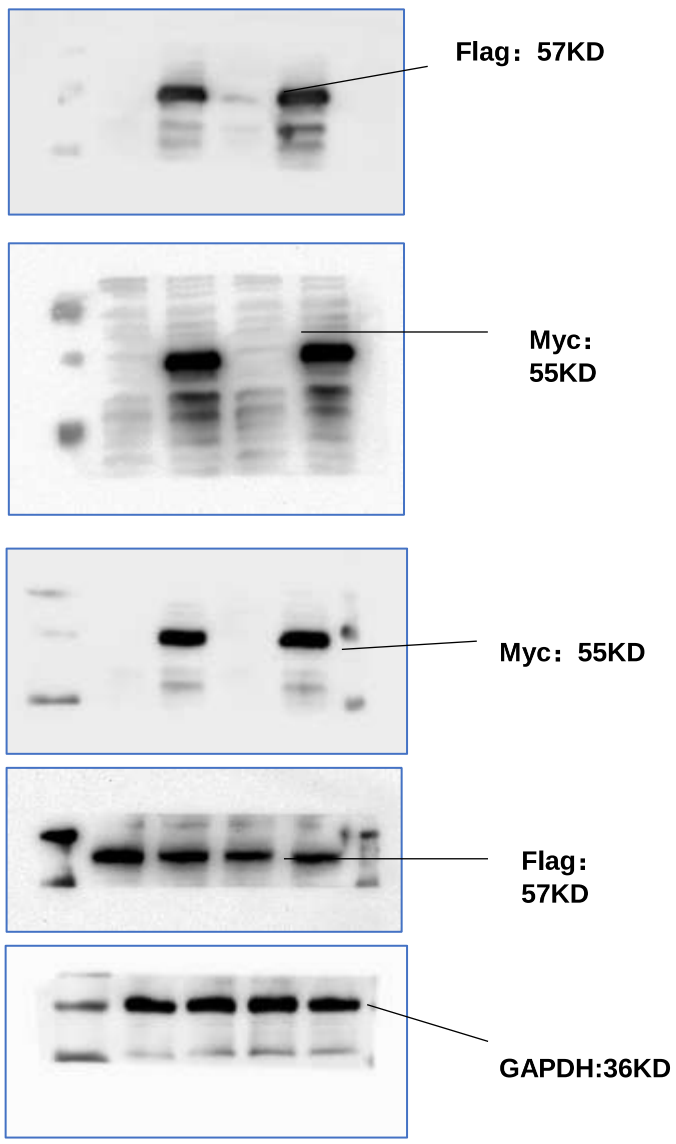

Figure 4e

on fig：

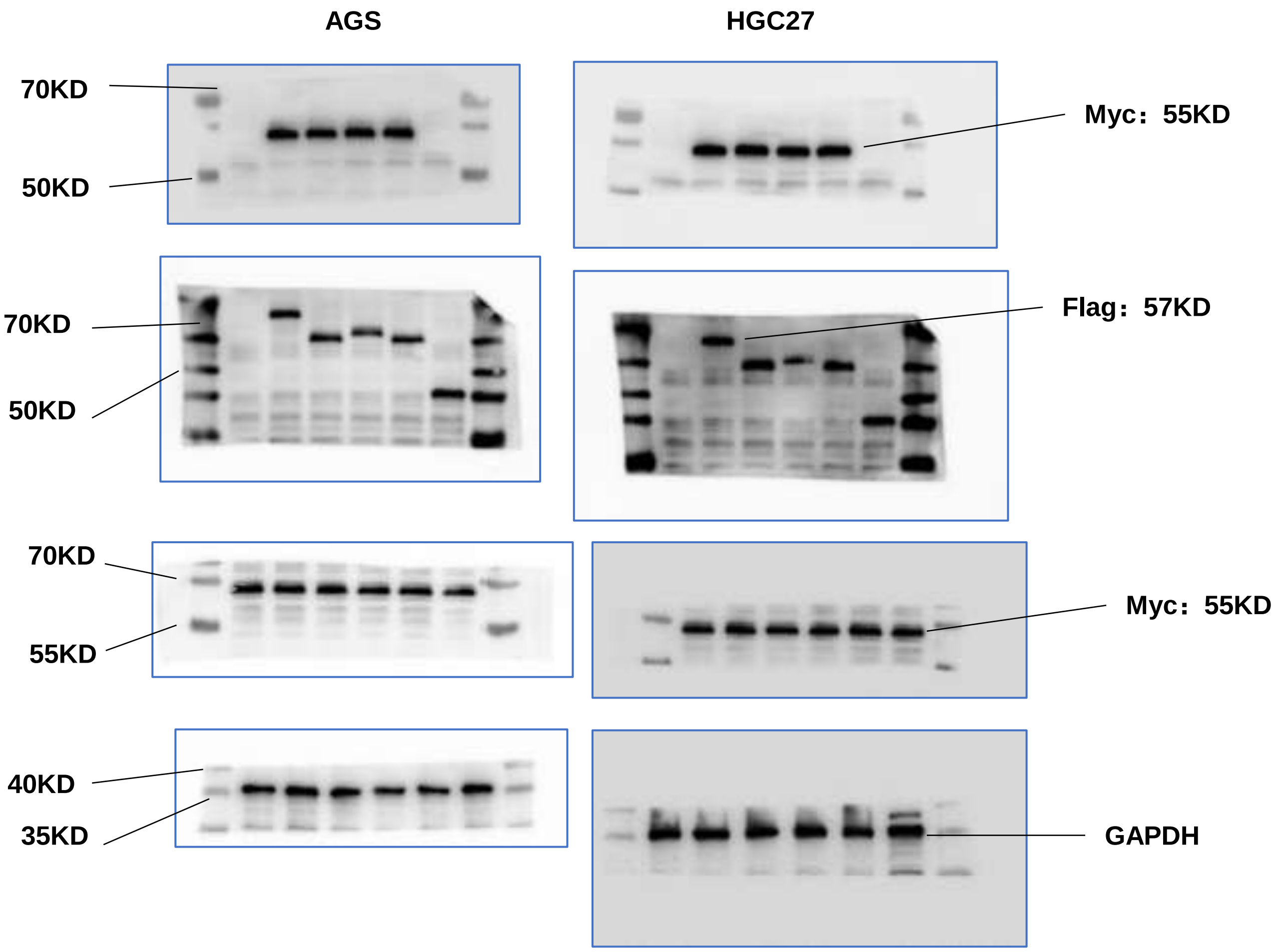

Figure 4e Repeat：

1：

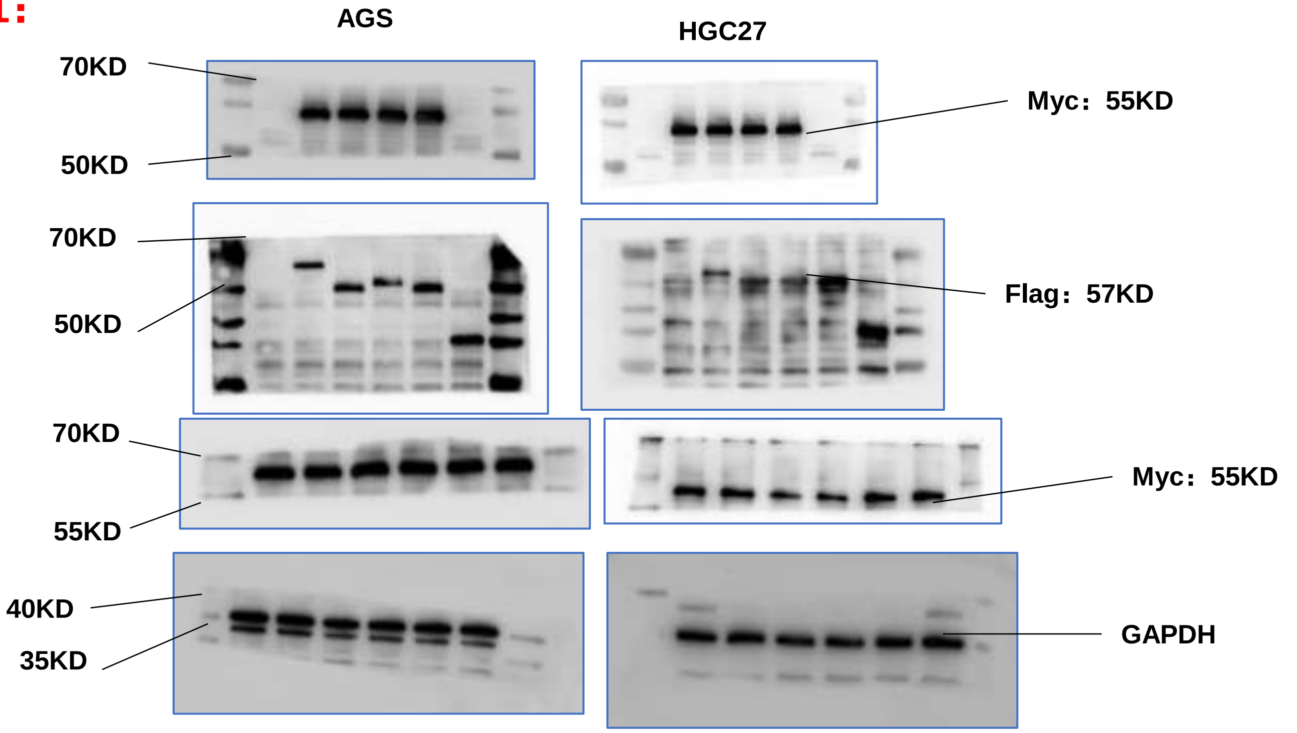

2：

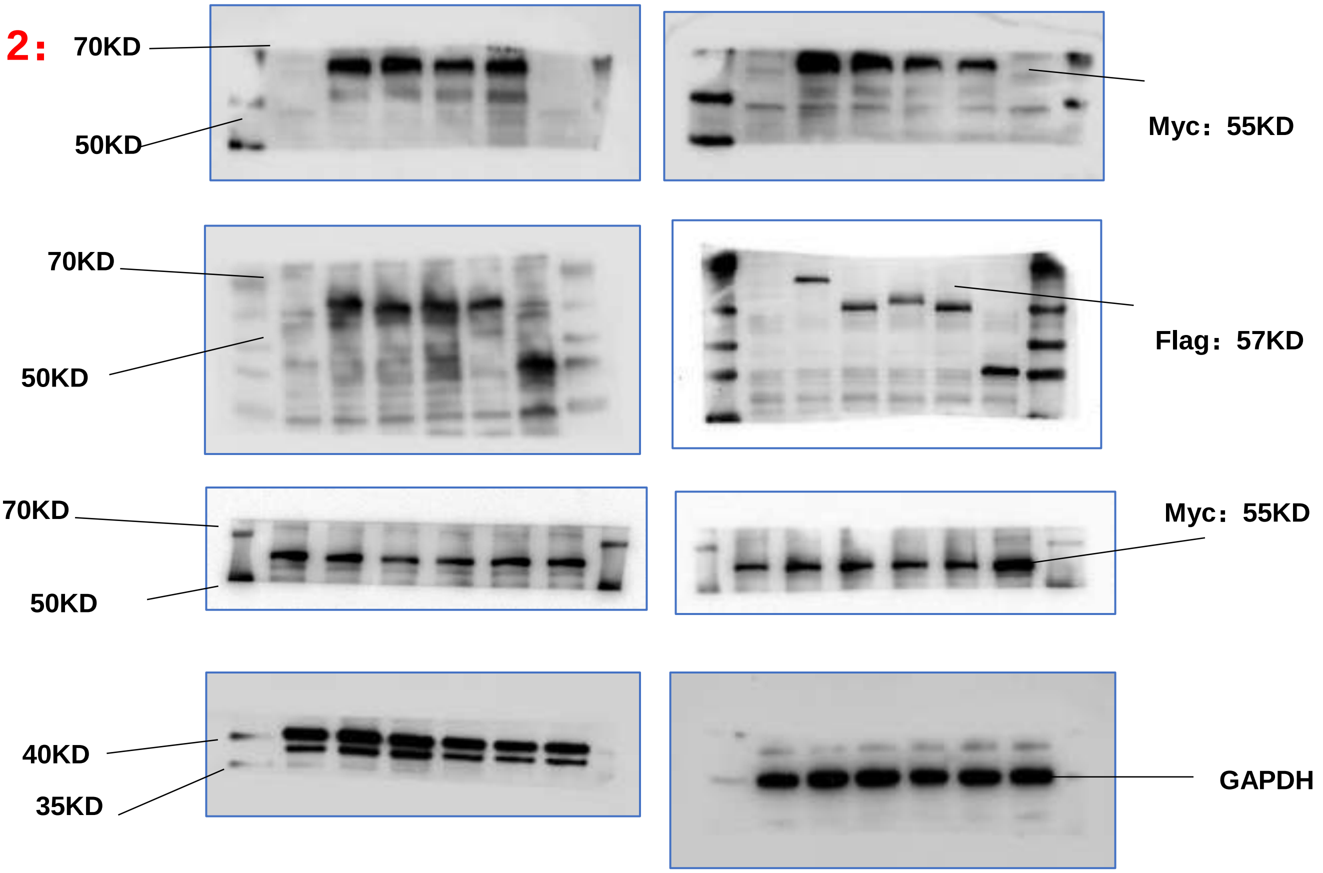

Figure 5c

on fig：

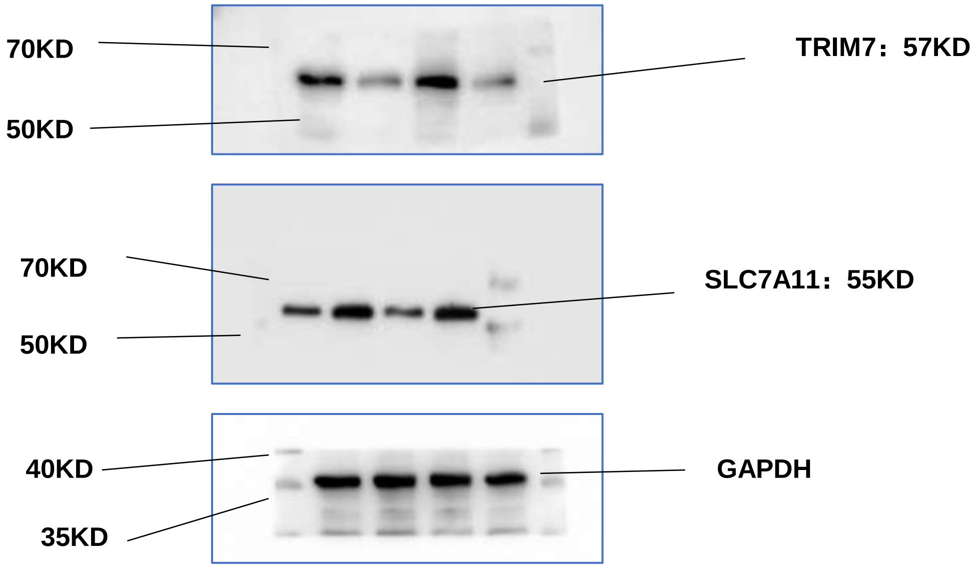

Repeat：

1：

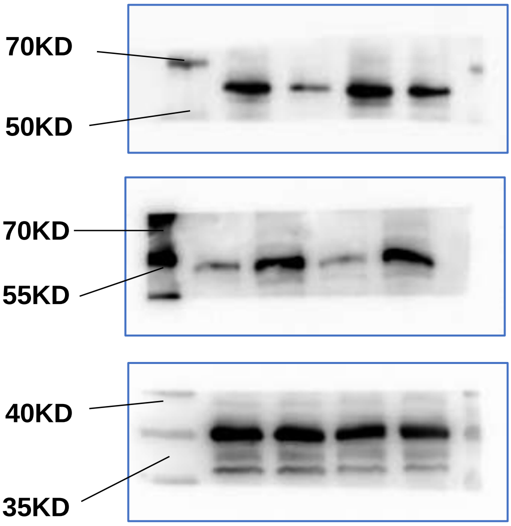

2：

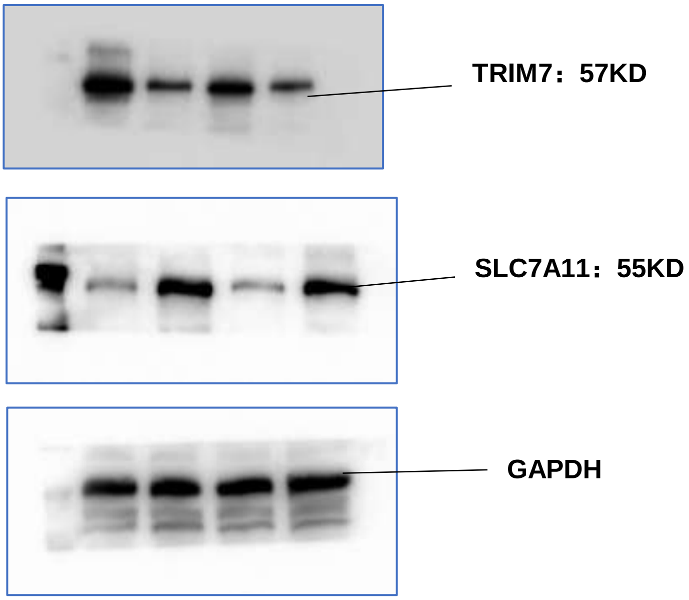

Figure 5d

on fig：

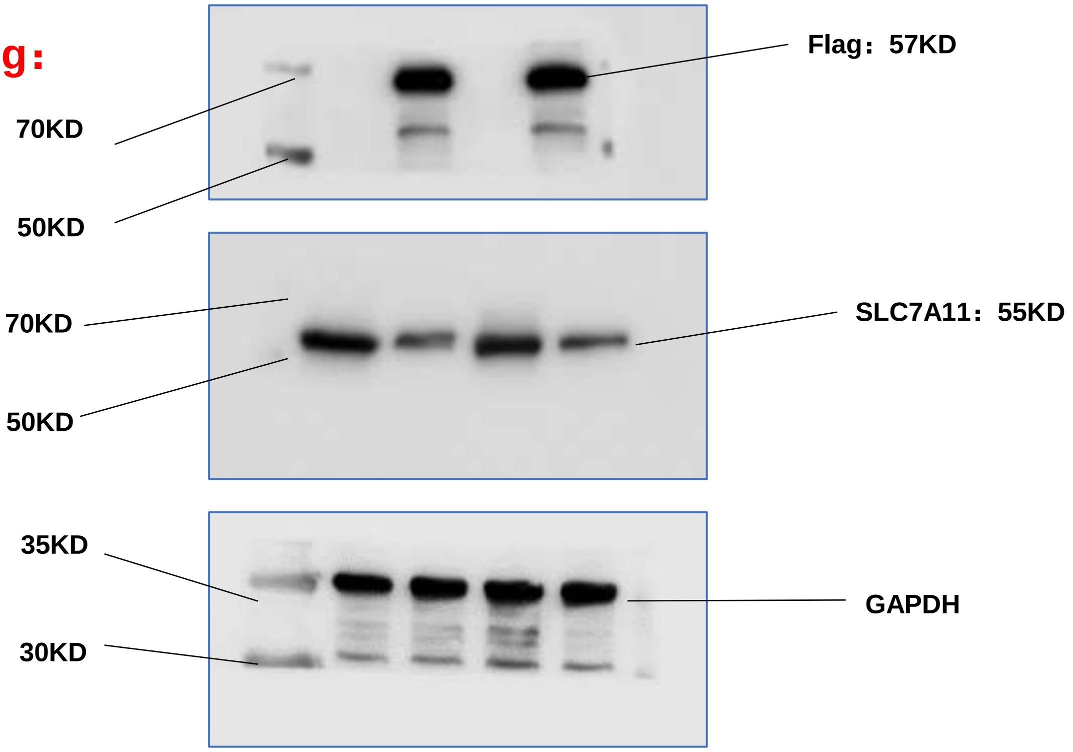

Repeat：

1：

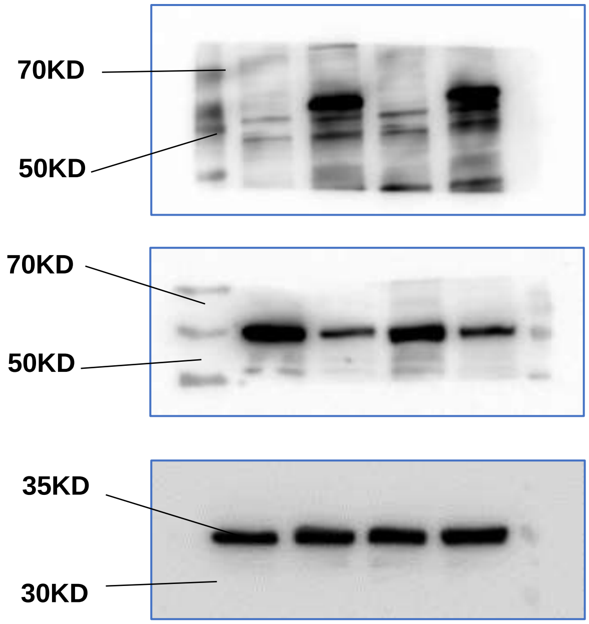

2：

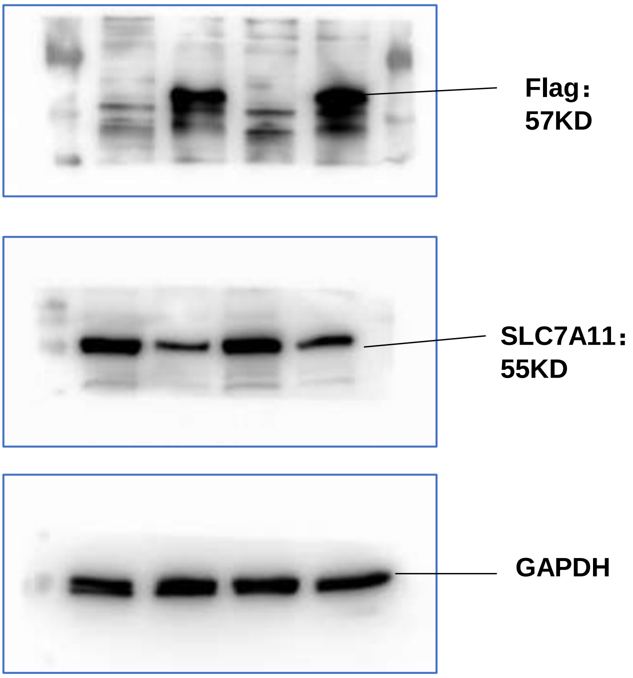

Figure 5e

on fig:

AGS

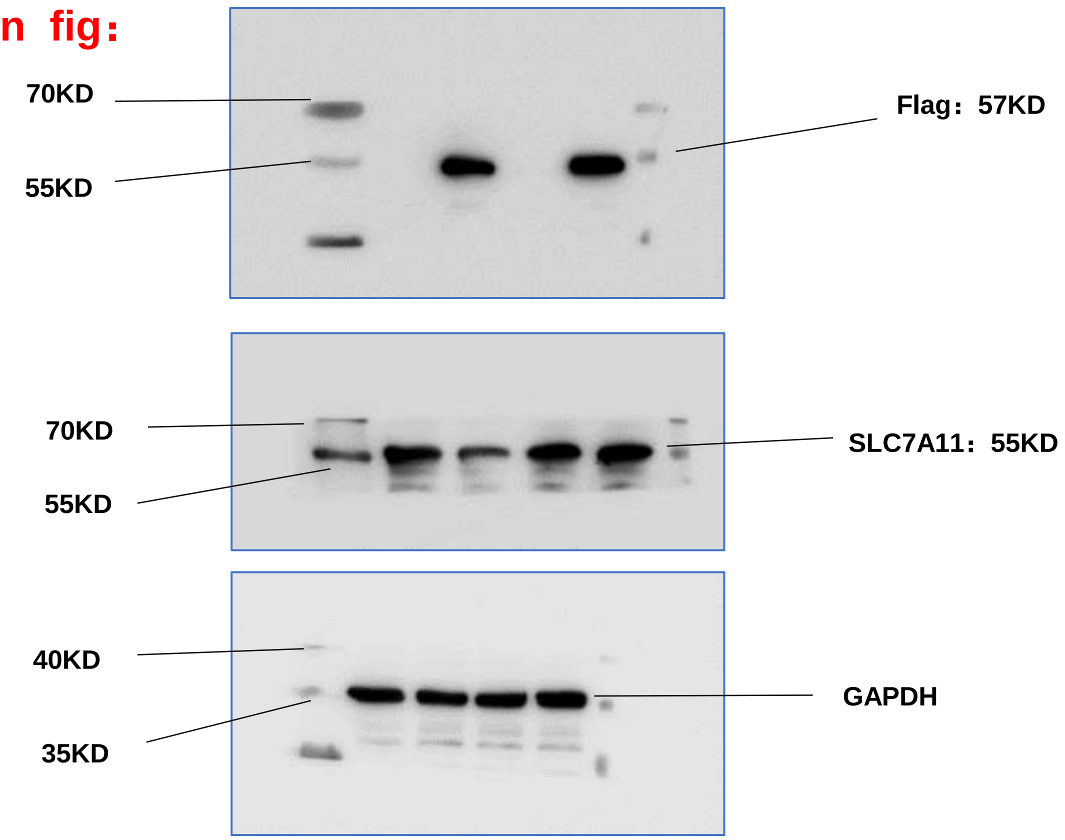

HGC27

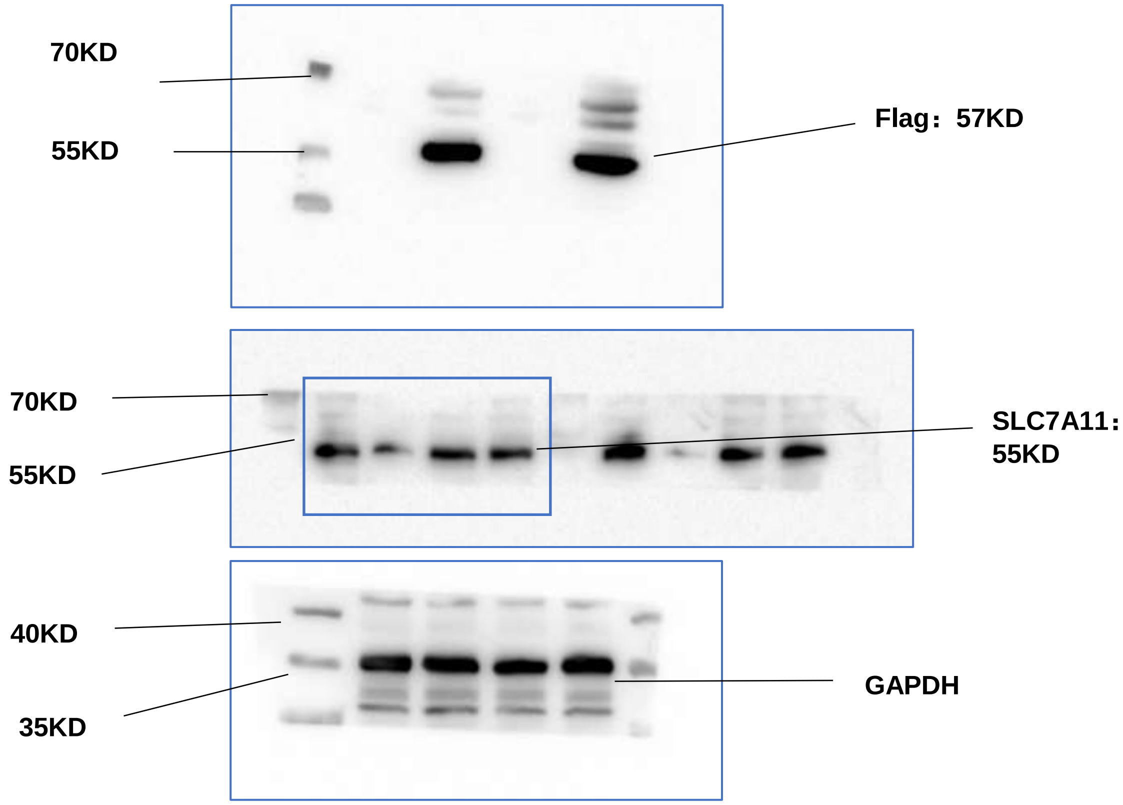

Figure 5e Repeat：

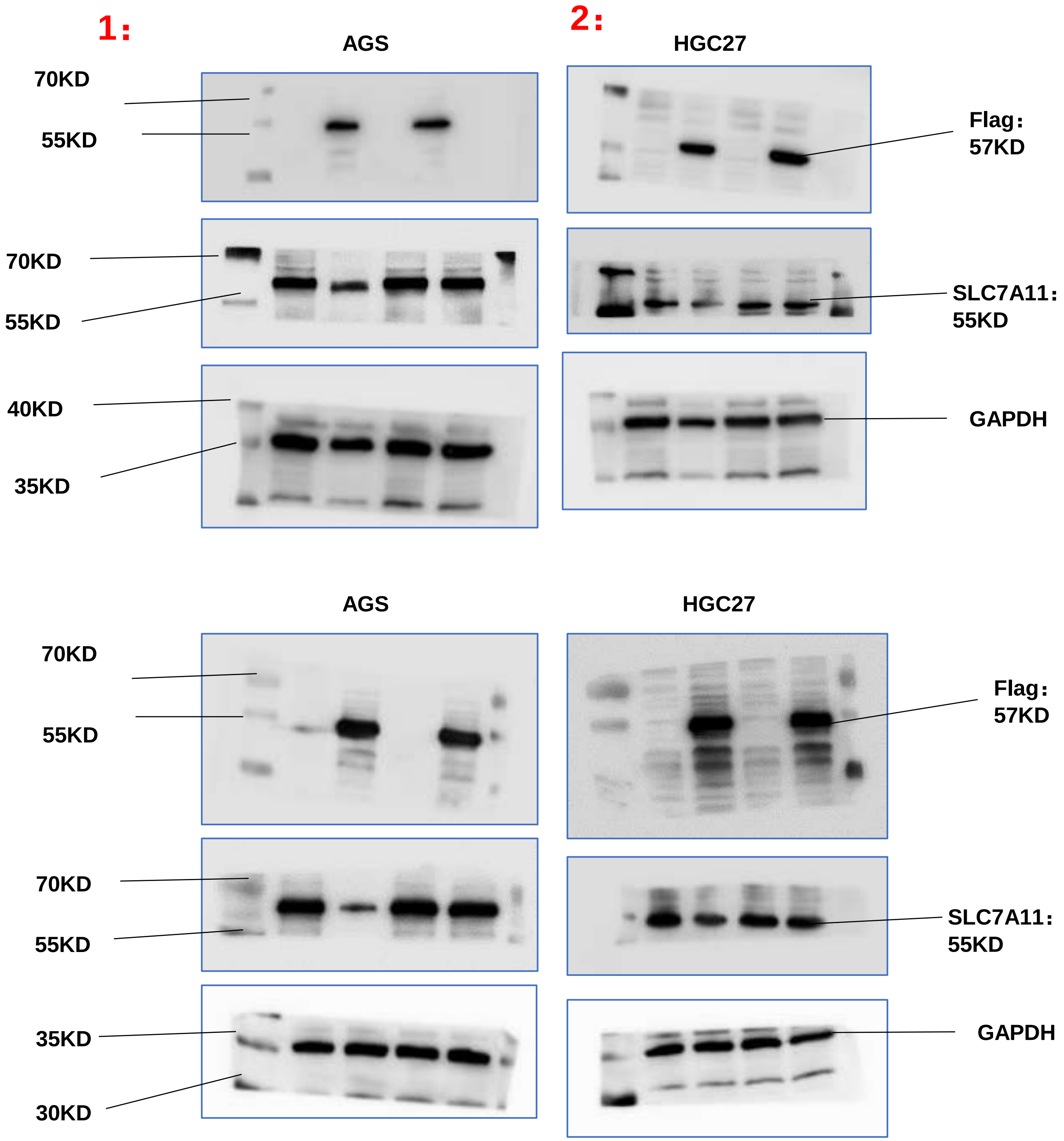

Figure 5f

on fig：

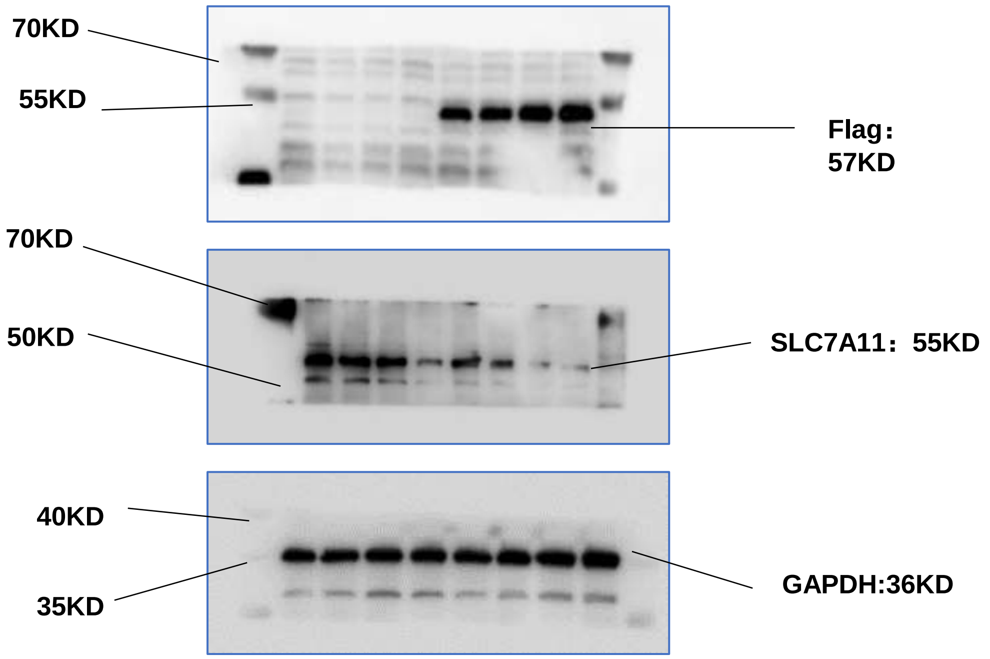

Repeat：

1：

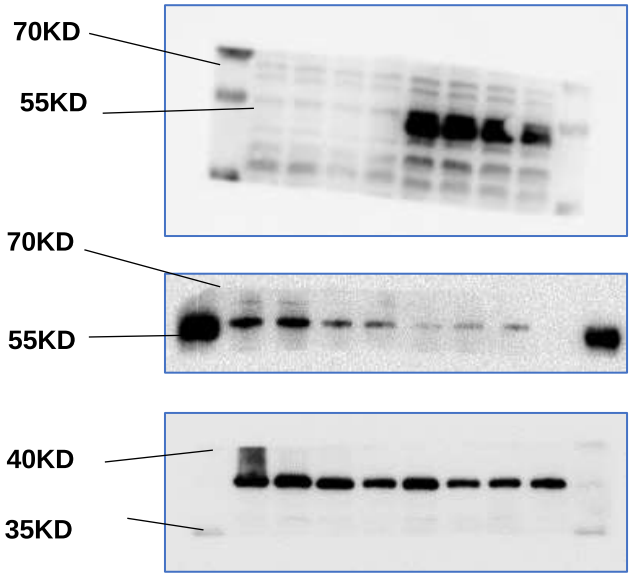

2：

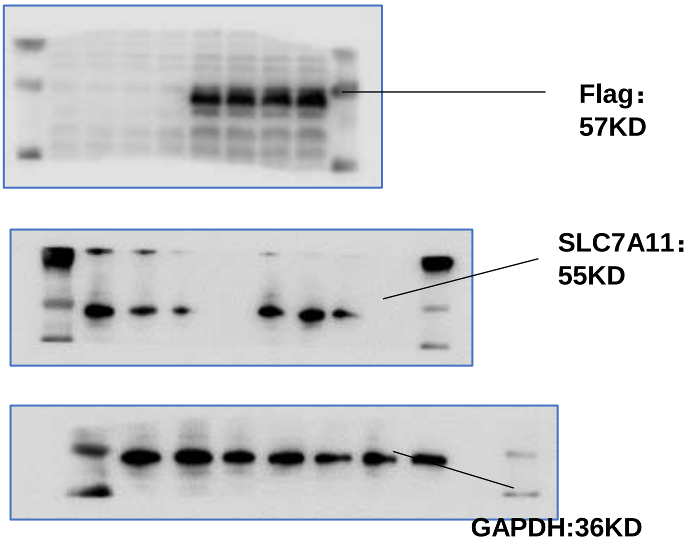

Figure 5g

on fig：

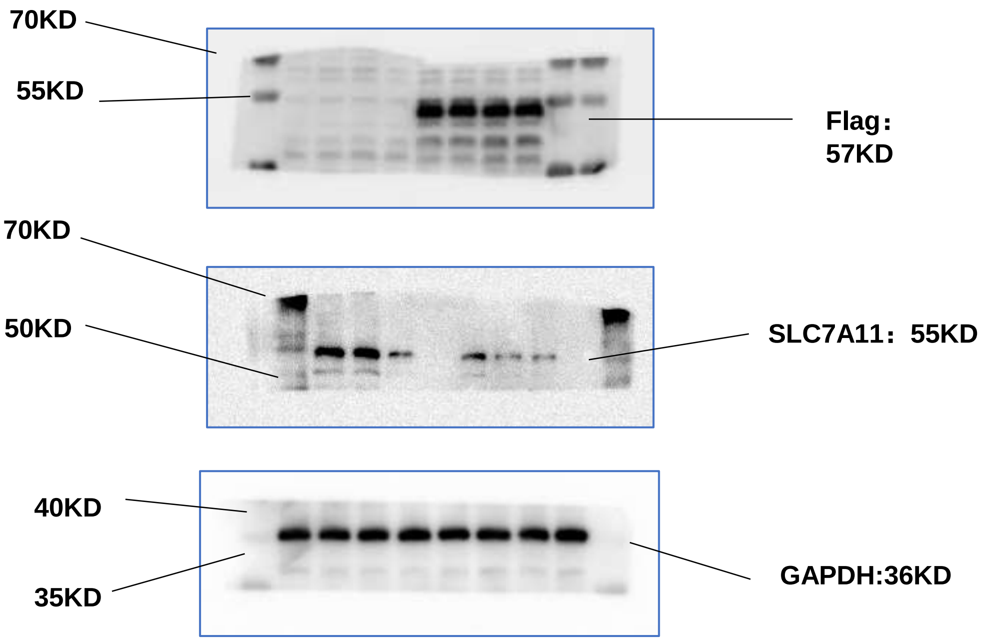

Repeat：

1：

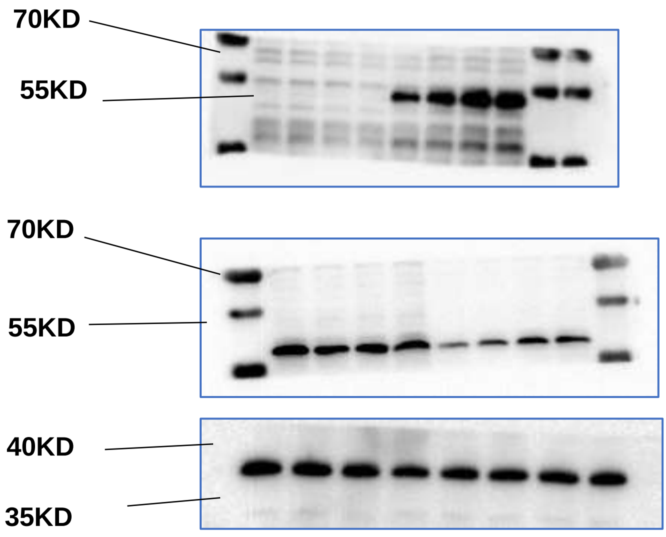

2：

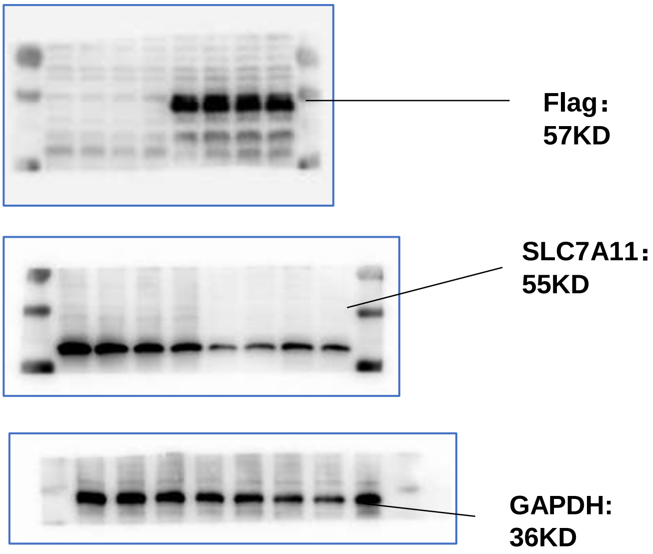

Figure 5h

on fig：

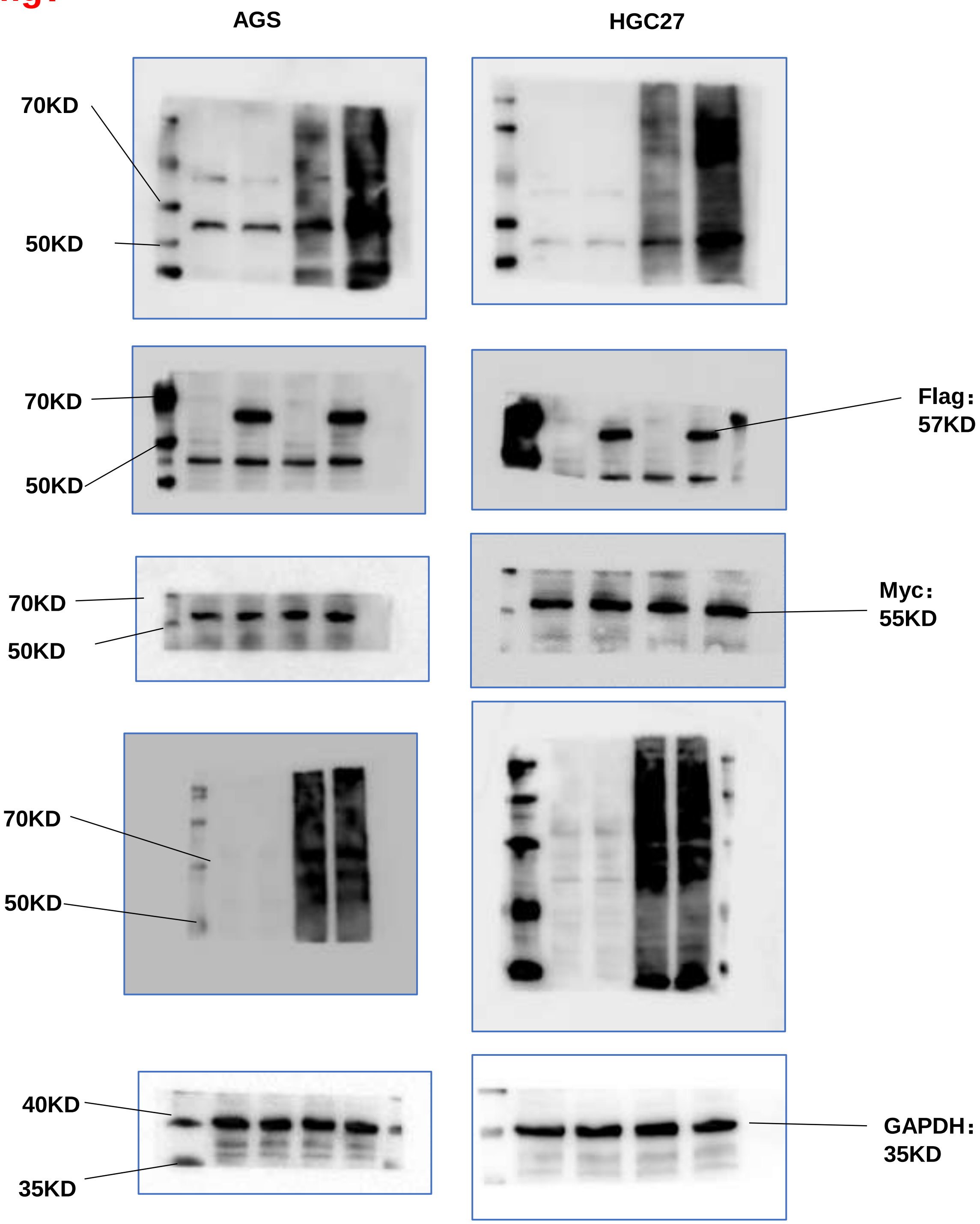

Figure 5h Repeat：

1：

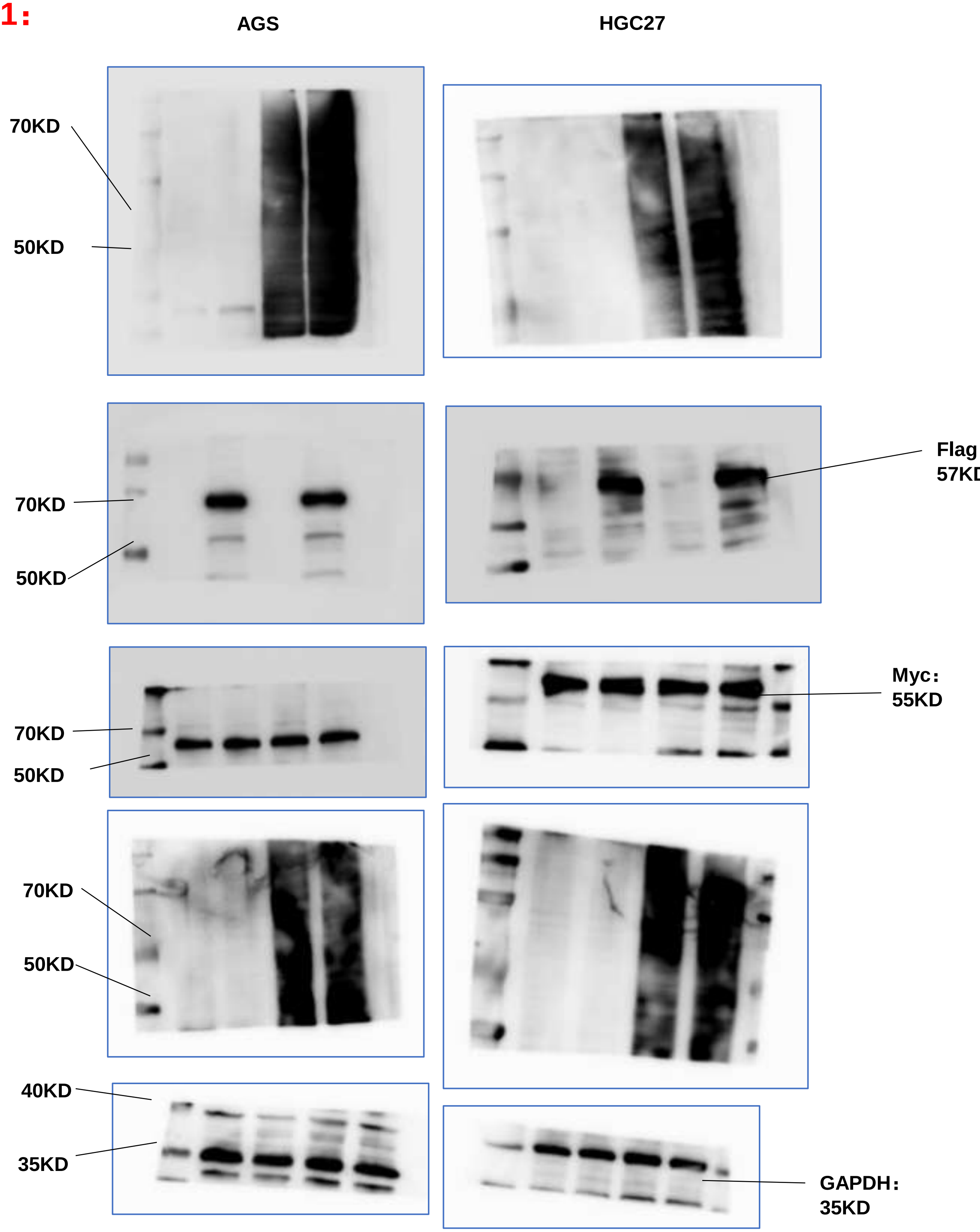

Figure 5h Repeat：

2：

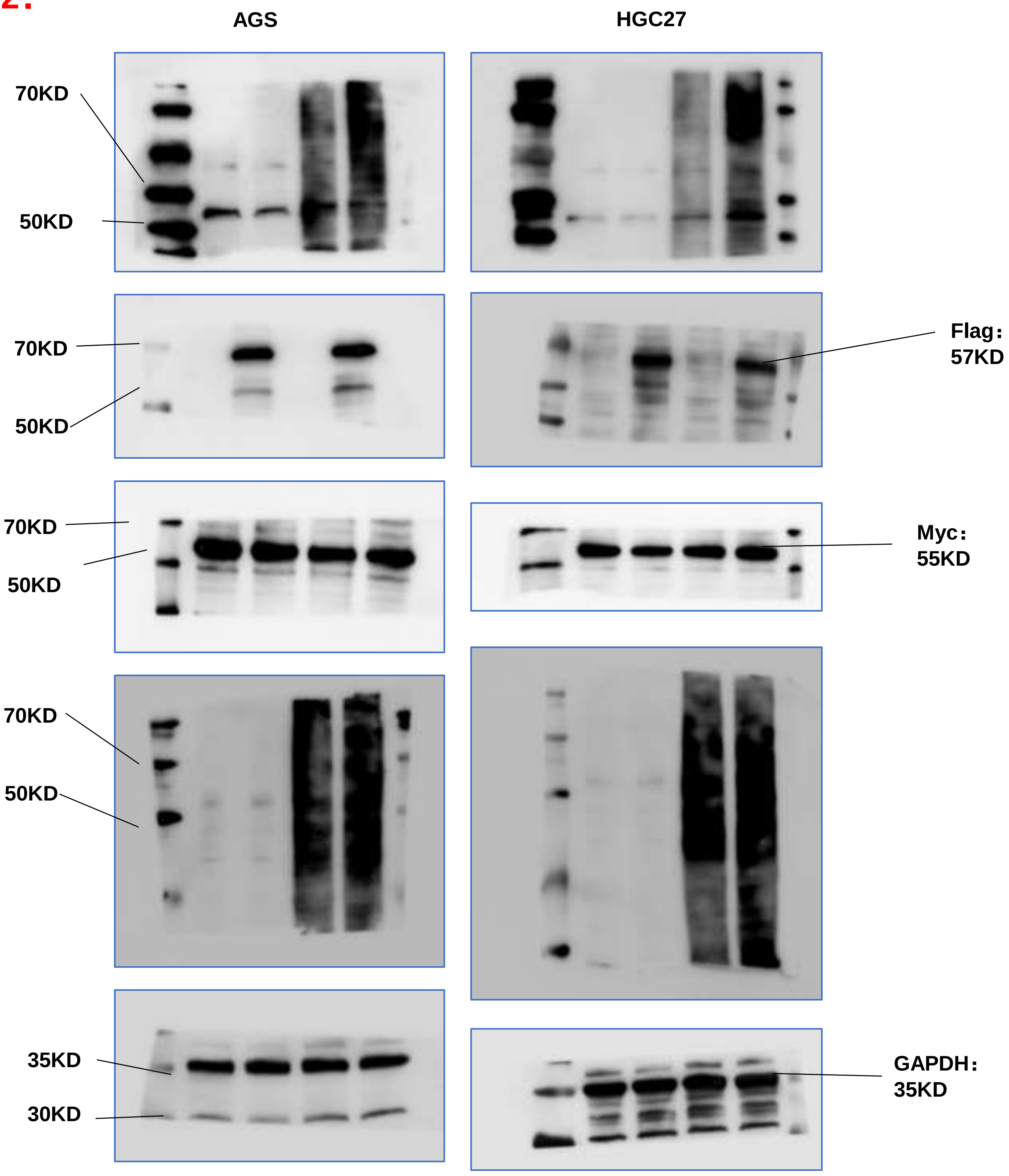

Figure 5i

on fig：

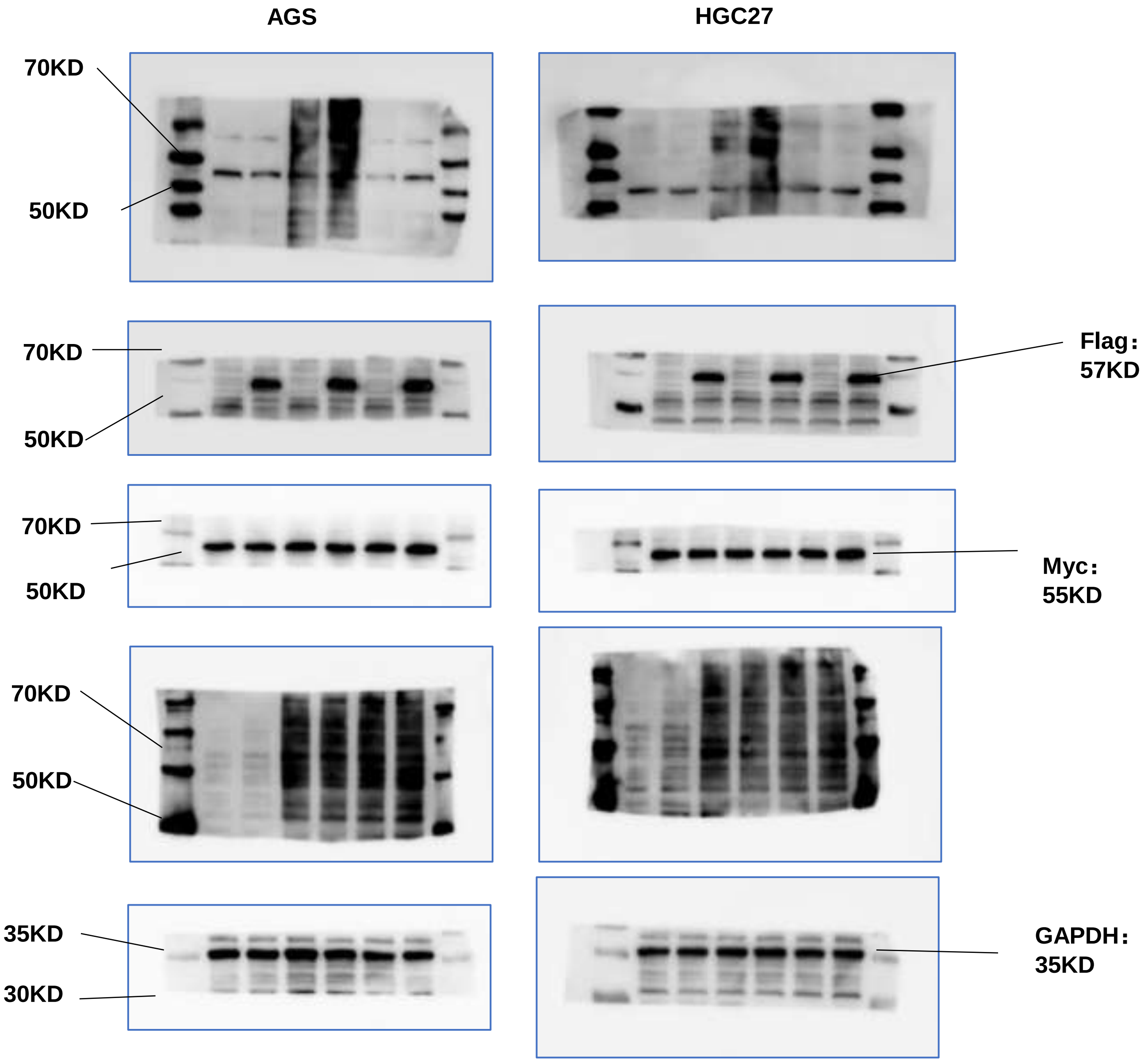

Figure 5i   Repeat：

1：

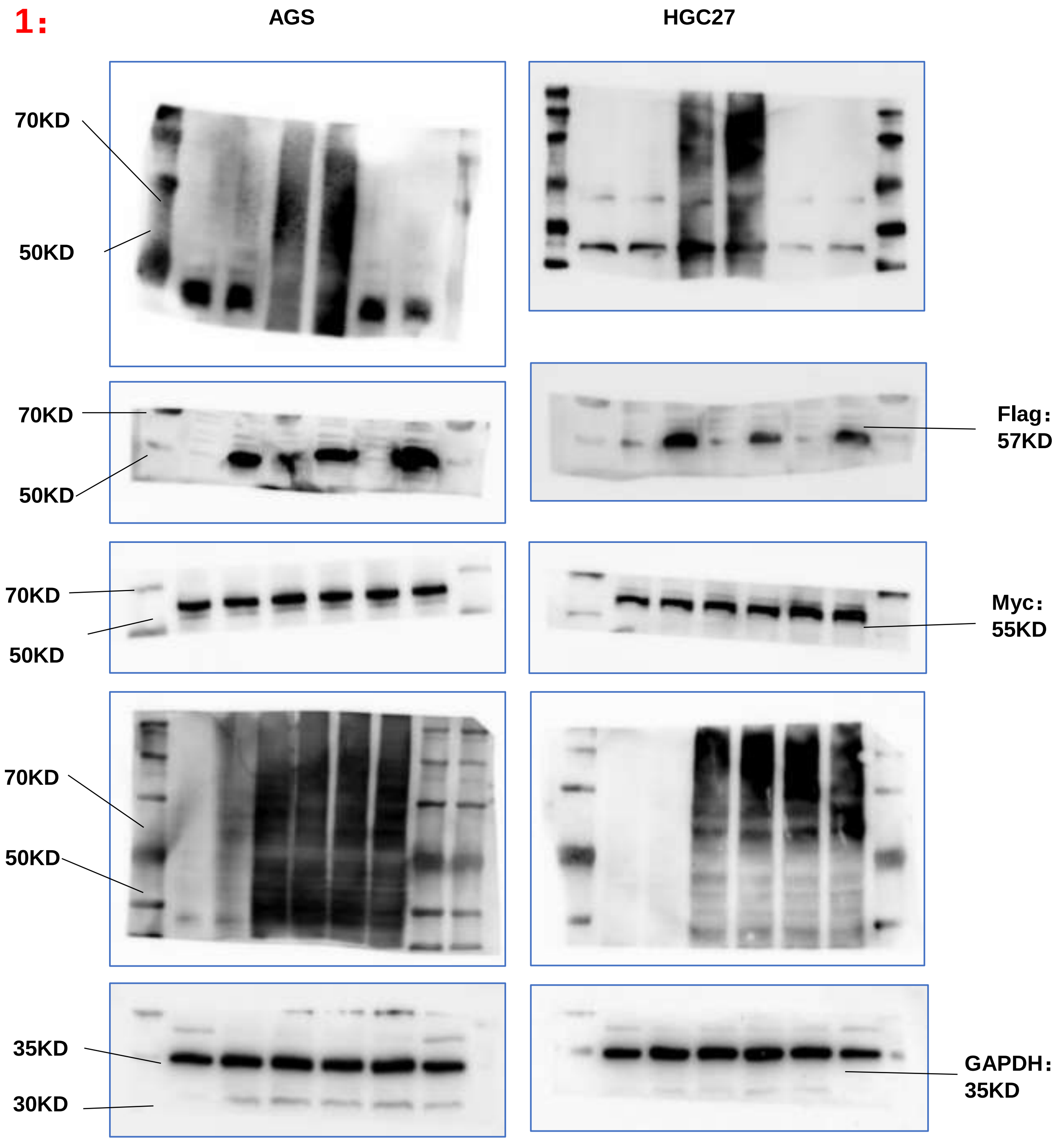

Figure 5i   Repeat：

2：

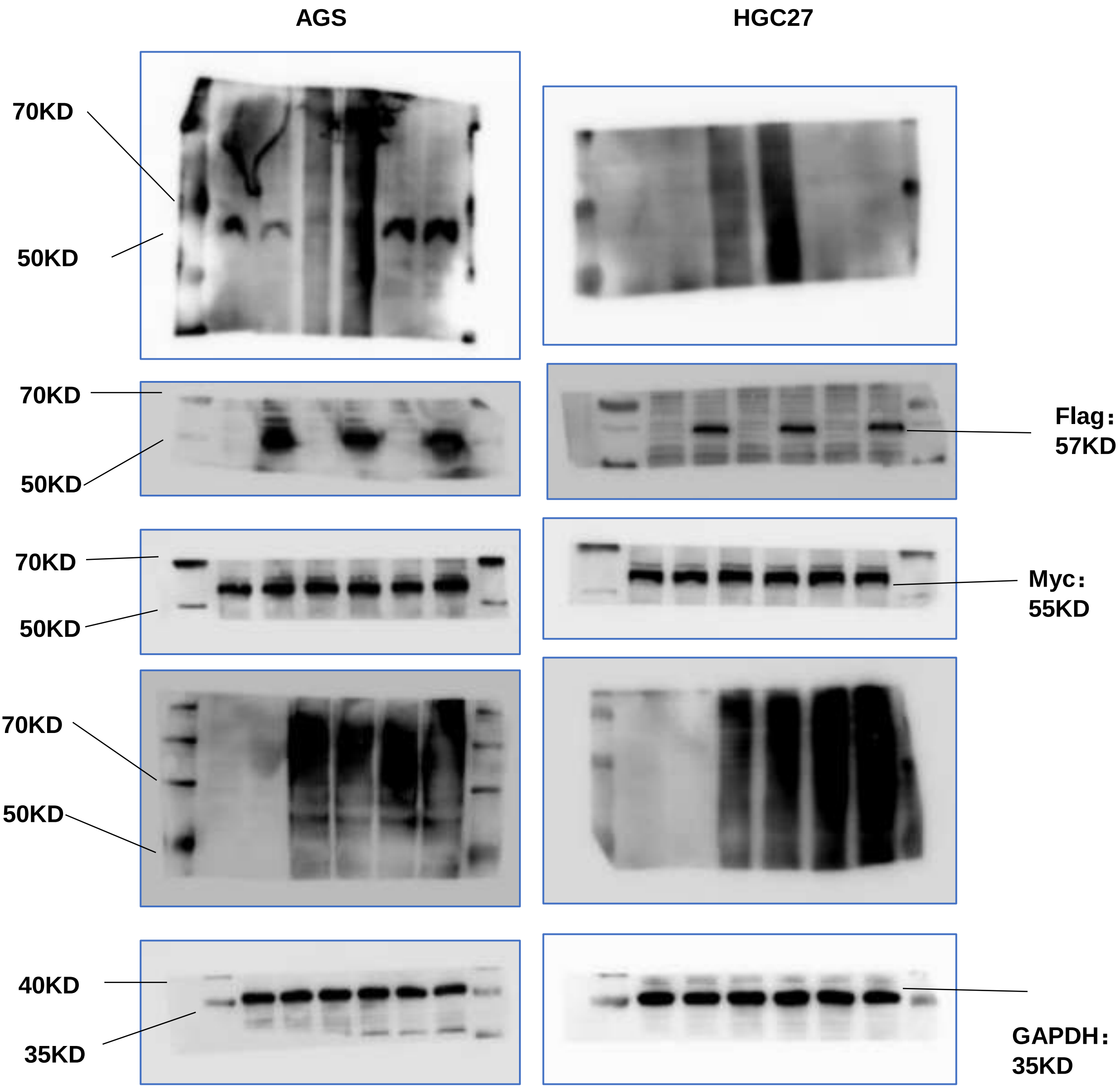

Figure 6a

on fig：

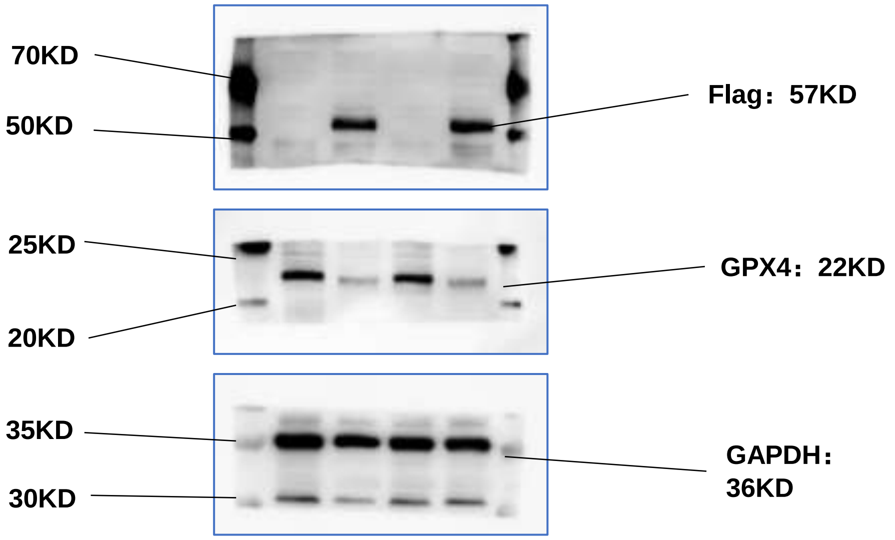

Repeat：

1：

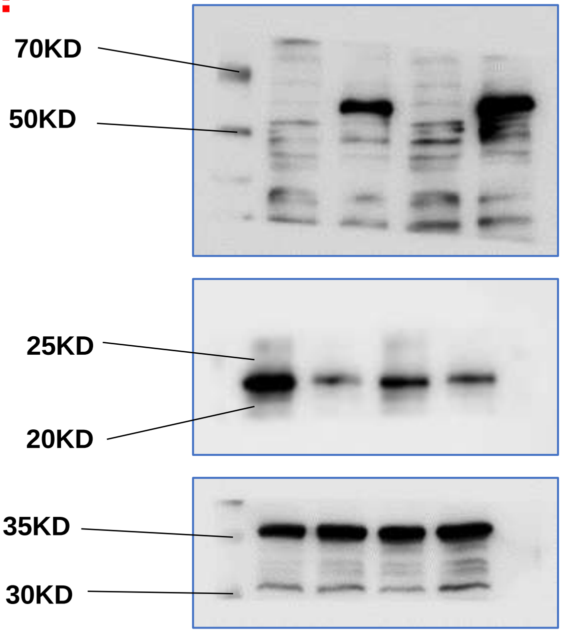

2：

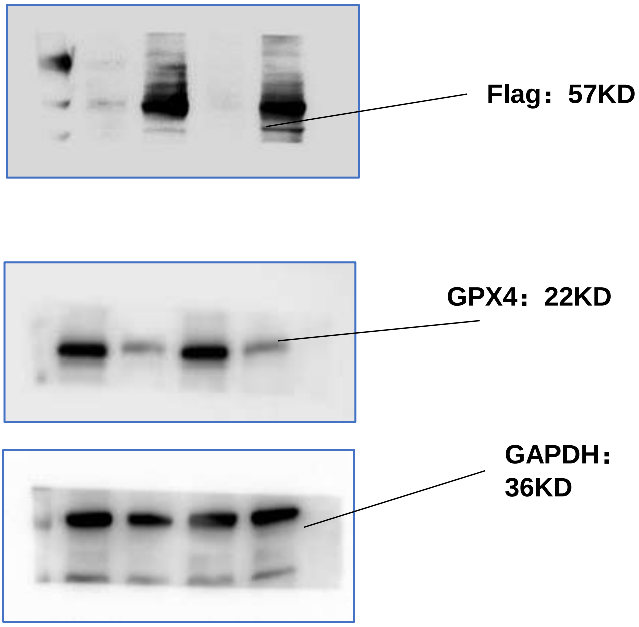

Figure 6b

on fig：

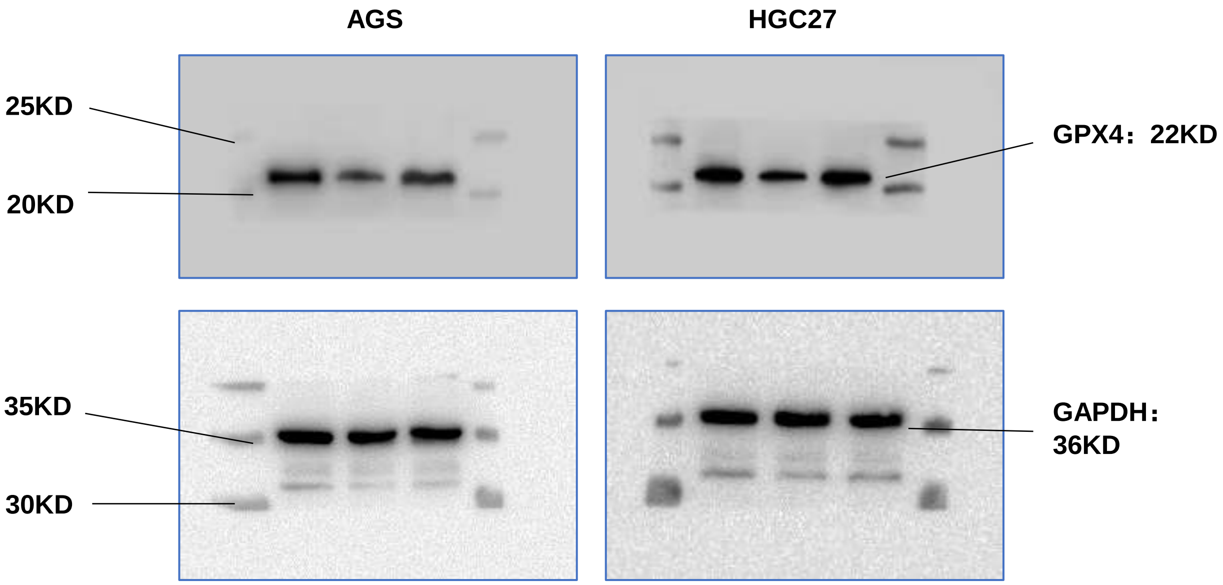

Figure 6b Repeat:

1:

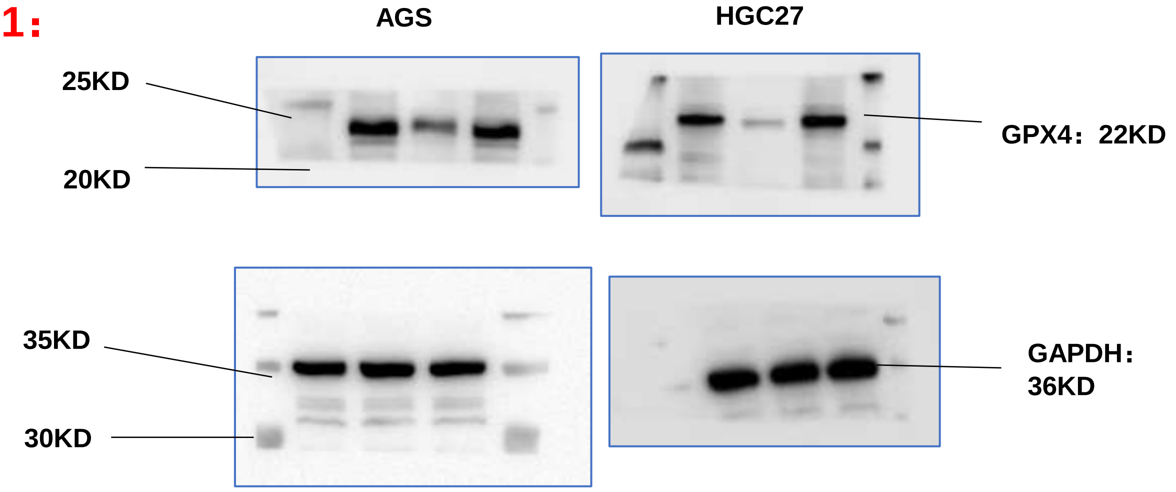

2:

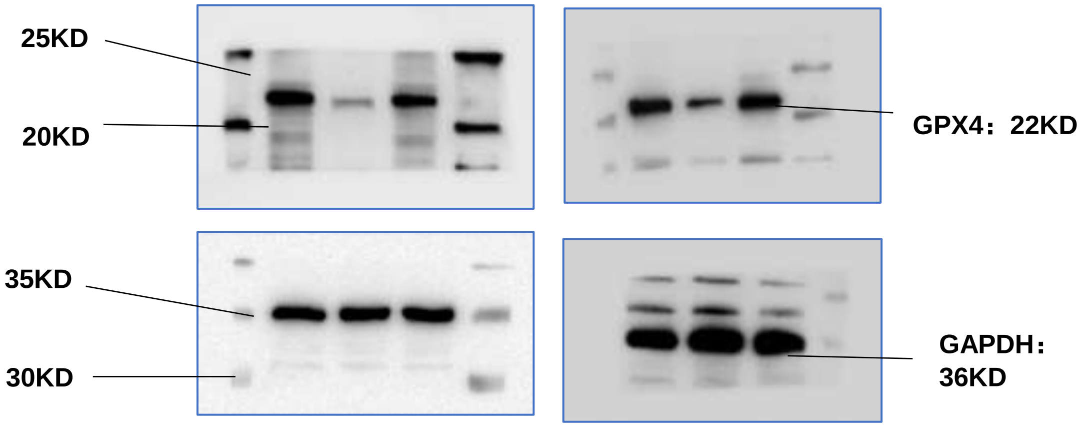

Supplementary Figure 1a

on fig：

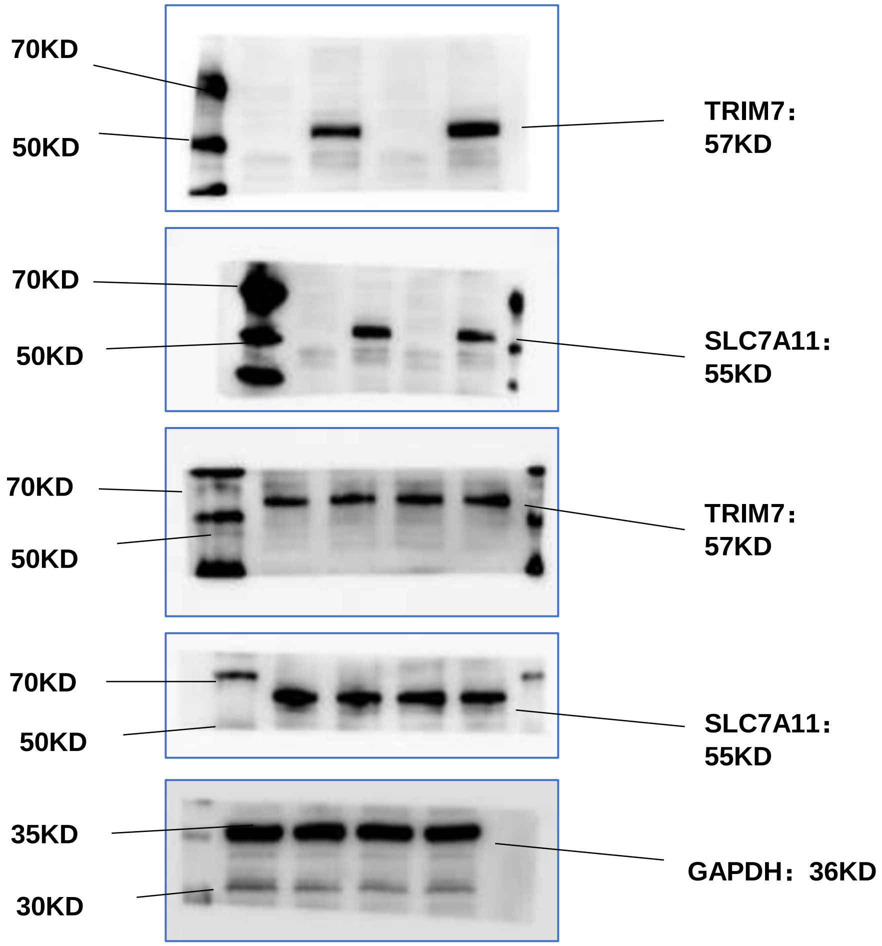

Supplementary Figure 1a **Repeat：**

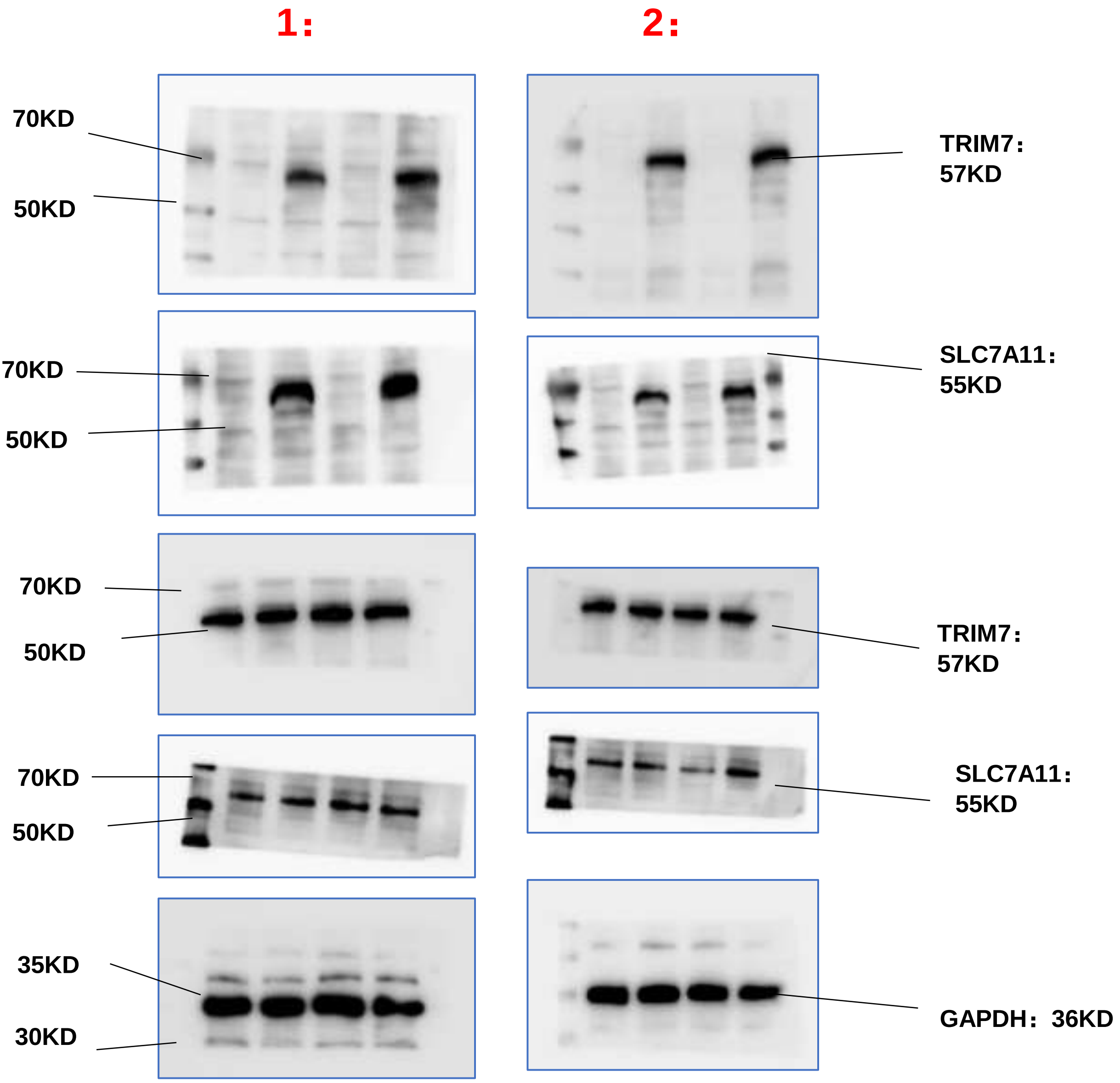

Supplementary Figure 1b

on fig：

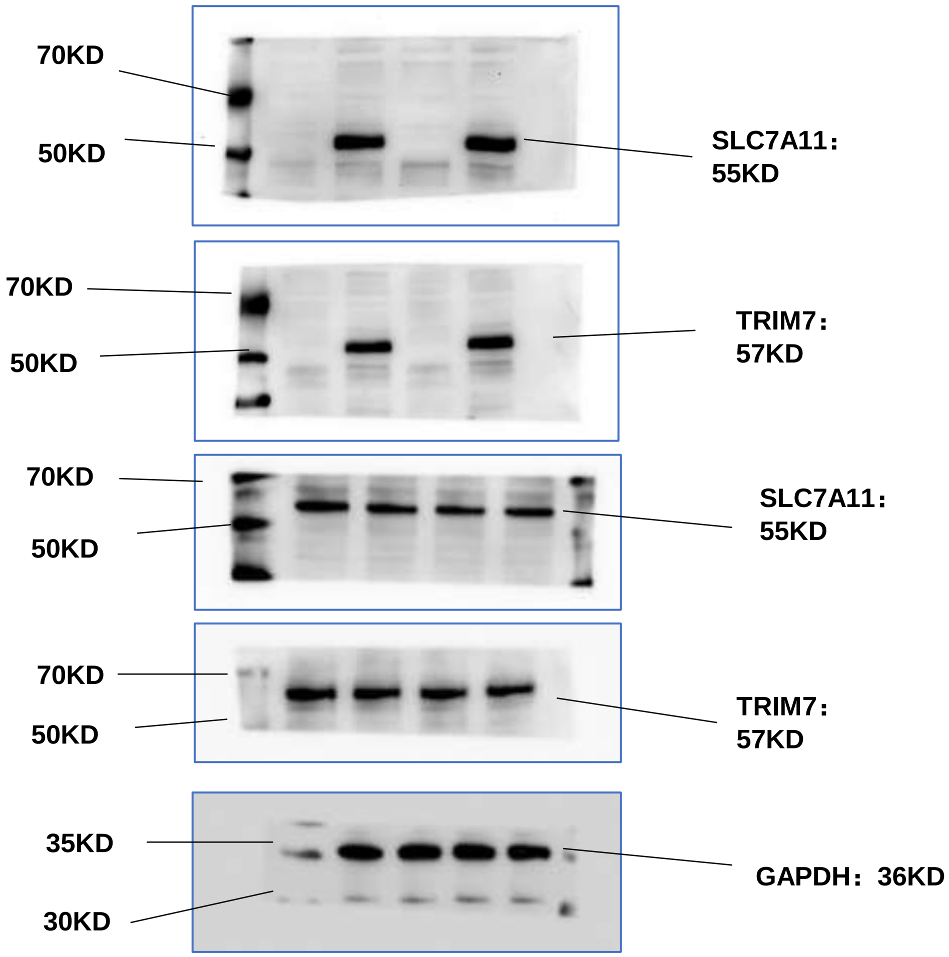

Supplementary Figure 1b Repeat：

1： 2：

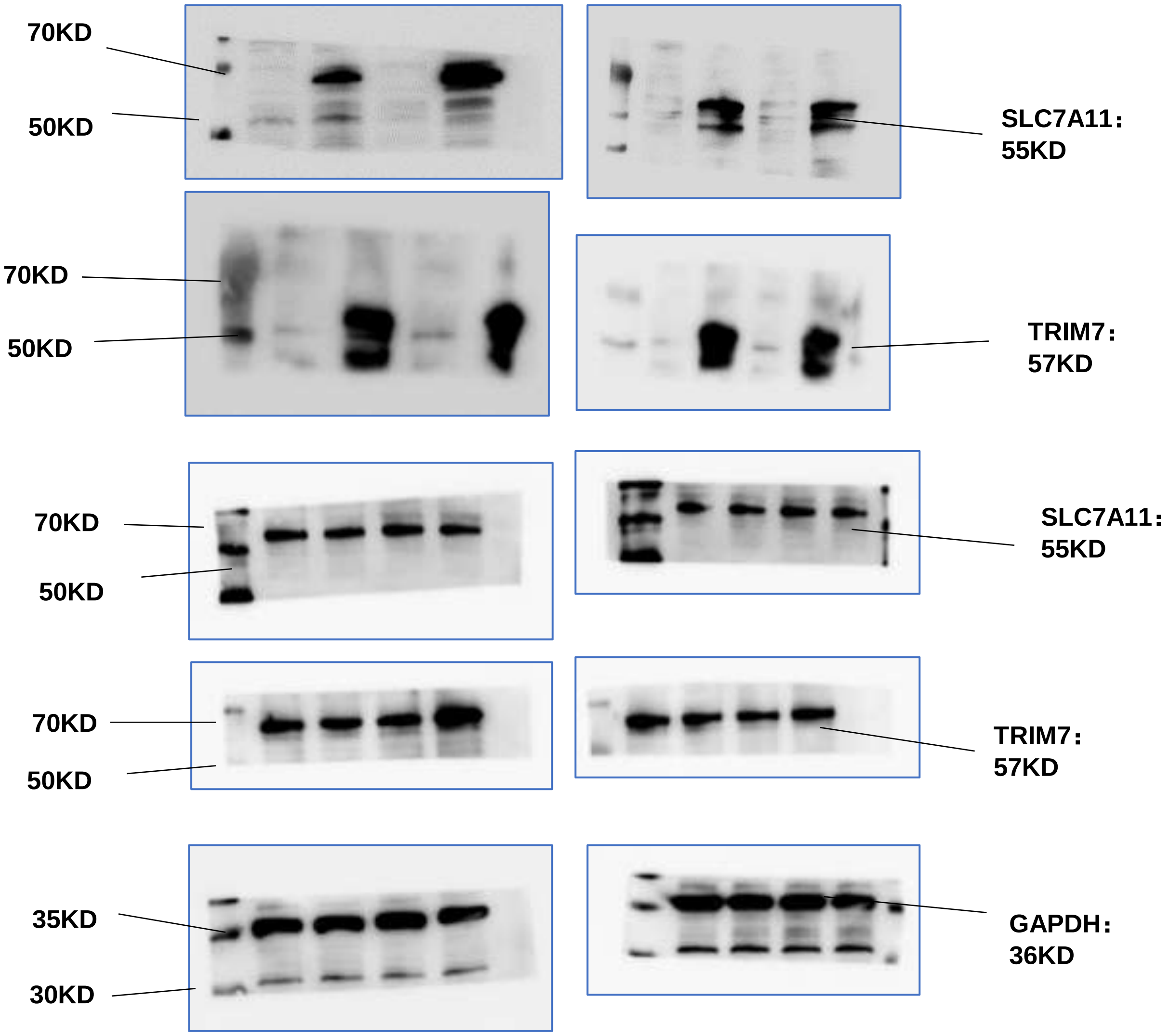

Supplementary Figure 2a

on fig：

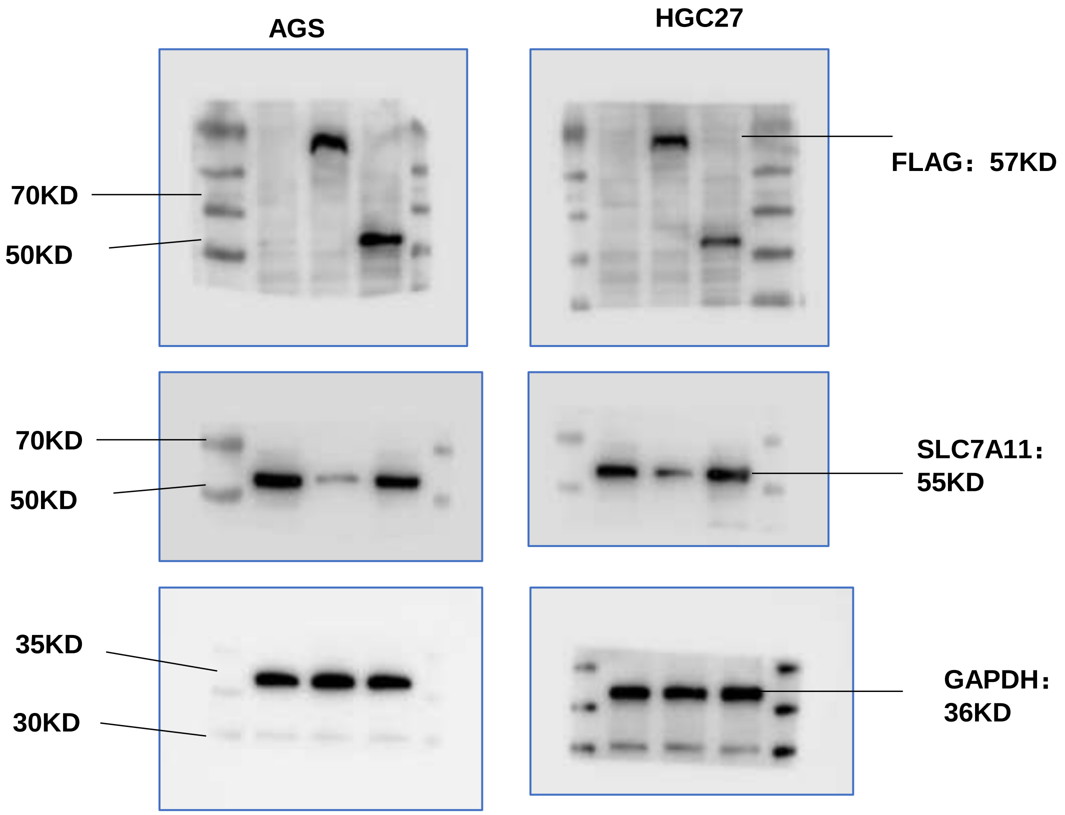

Supplementary Figure 2a Repeat：

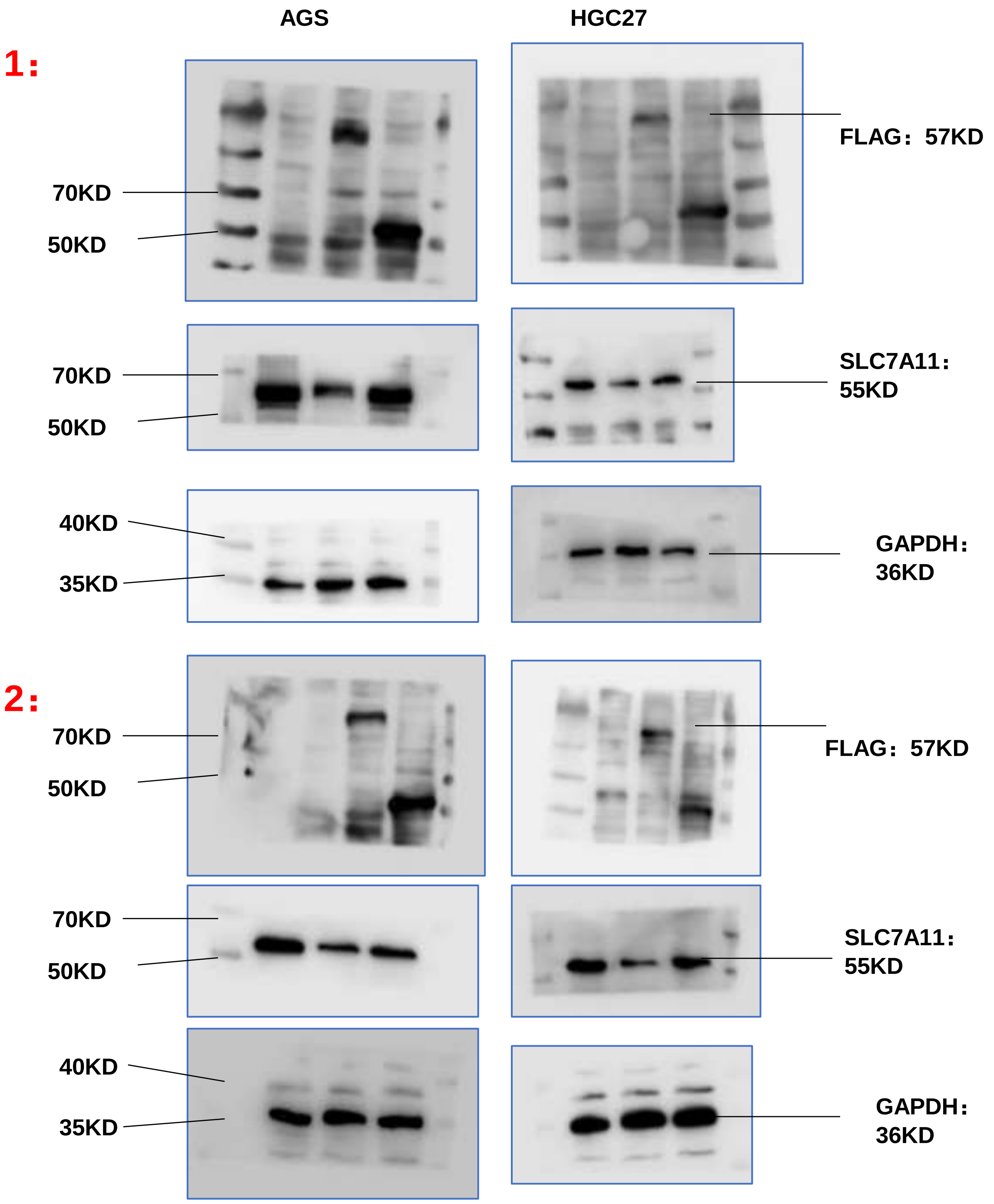

Supplement: Supplementary file 1 — Supplementary Figures. [file 41598_2024_56746_MOESM1_ESM.pdf]
